# High and intermediate temperature sodium–sulfur batteries for energy storage: development, challenges and perspectives

**DOI:** 10.1039/c8ra08658c

**Published:** 2019-02-14

**Authors:** Georgios Nikiforidis, M. C. M. van de Sanden, Michail N. Tsampas

**Affiliations:** Dutch Institute for Fundamental Energy Research (DIFFER) De Zaale 20 Eindhoven 5612AJ The Netherlands; Organic Bioelectronics Lab, Biological and Environmental Science and Engineering Division, King Abdullah University of Science and Technology (KAUST) Saudi Arabia georgios.nikiforidis@kaust.edu.sa; Department of Applied Physics, Eindhoven University of Technology 5600 MB Eindhoven The Netherlands

## Abstract

In view of the burgeoning demand for energy storage stemming largely from the growing renewable energy sector, the prospects of high (>300 °C), intermediate (100–200 °C) and room temperature (25–60 °C) battery systems are encouraging. Metal sulfur batteries are an attractive choice since the sulfur cathode is abundant and offers an extremely high theoretical capacity of 1672 mA h g^−1^ upon complete discharge. Sodium also has high natural abundance and a respectable electrochemical reduction potential (−2.71 V *vs.* standard hydrogen electrode). Combining these two abundant elements as raw materials in an energy storage context leads to the sodium–sulfur battery (NaS). This review focuses solely on the progress, prospects and challenges of the high and intermediate temperature NaS secondary batteries (HT and IT NaS) as a whole. The already established HT NaS can be further improved in terms of energy density and safety record. The IT NaS takes advantage of the lower operating temperature to lower manufacturing and potentially operating costs whilst creating a safer environment. A thorough technical discussion on the building blocks of these two battery systems is discussed here, including electrolyte, separators, cell configuration, electrochemical reactions that take place under the different operating conditions and ways to monitor and comprehend the physicochemical and electrochemical processes under these temperatures. Furthermore, a brief summary of the work conducted on the room temperature (RT) NaS system is given seeking to couple the knowledge in this field with the one at elevated temperatures. Finally, future perspectives are discussed along with ways to effectively handle the technical challenges presented for this electrochemical energy storage system.

## Abstract 2

This comprehensive review focuses on the progress, prospects and challenges of the high and intermediate NaS secondary batteries (HT and IT NaS) as a whole.

## Graphical abstract

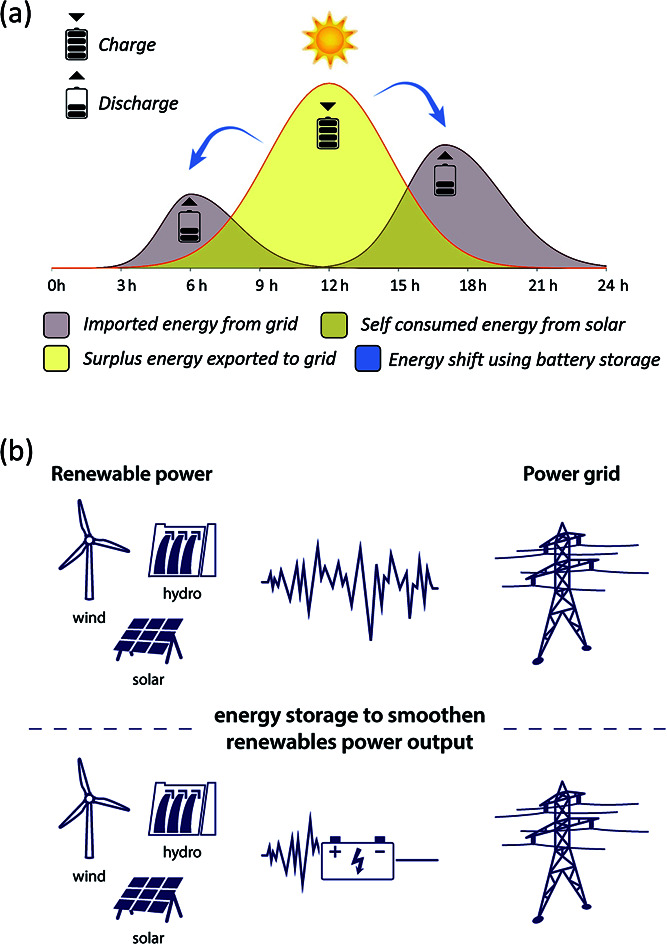

## 1 Energy storage outlook

Renewable energy is derived from resources that are replenished naturally on a human timescale. They differ from fossil fuels in their diversity and abundance and most importantly they do not produce greenhouse gases. Some of these sources are topographically limited even though they produce a constant supply of electricity, such as geothermal and hydropower, whilst others produce electricity in an intermittent manner (*i.e.* wind and solar). The quickly dropping cost of wind and solar electricity generation is reflected by the competitive levelized costs of electricity (LCOE) to that of fossil fuel generation, nuclear power plants and combined cycle gas turbines in several modernized countries around the world including Germany, Australia, Spain and the United States.^1,2^ The LCOE from electricity to solar is set to drop 66% by 2040 while onshore and offshore wind levelized costs will decline by 47 and 71%, respectively owing to economies of scale, experience and competition.

Replacing the incumbent energy model of centralized fossil fuel electricity with a more decentralized model that will be based on high penetration rates of intermitting renewable energy carries numerous challenges. Together with lower power rating batteries, the renewable energy penetration in advanced and developing economies is expected to increase significantly. Examples include Germany (74%), U.S.A (55%), China (49%) and India (49%).^3^ Above a penetration rate of 30%, intermittent renewable energy with no energy storage can prompt a mismatch between supply and demand leading to low power quality, network constraints and renewable energy curtailment.^4^ Therefore, versatile energy storage technologies that can readily provide demand and peak reduction, network congestion relief and infrastructure deferral, rapid frequency, superior power quality and voltage response along with seasonal storage are indispensable.^5^ This task can be fulfilled by electrical energy storage systems (EES).^6^

EES can be classified with regards to their energy form used to mechanical, chemical, electrochemical, thermal, and electrical. Key parameters of these systems include the ground facilities, interaction with existing uses of gas, optimal (electro)chemical processes, safety, reliability and efficiency. Putting things in perspective, the current incumbent electrical energy storage technology is pumped hydro storage (PHS), a hydroelectric energy storage system that accounts for over 99% of installed storage capacity of electrical energy through its 270 sites globally reaching a total generating power capacity of 127 GW.^7,8^ PHS together with underground compressed air energy storage (CAES) carry the lowest installed cost per kW h (∼$100). The remaining 1% of the installed storage capacity is deployed by compressed air (41.5%) and a plethora of battery systems including LIB, SIB, NaS, advanced Pd–acid and Ni–Cd batteries, flywheel and redox flow batteries.^9^

The majority of the systems that constitute the non-pumped utility scale energy storage come under the electrochemical EES category (EEES). EEES yield higher efficiency compared to other ESS in terms of scalability, round-trip efficiency, calendar life, discharge time, weight and mobility of the system. At present, Battery Energy Storage Systems (BESS) hold a minor share in total battery capacity in stationary applications, yet rapid growth rates are forecasted with battery capacity increasing to 167 GW in 2030.^1^

**Fig. 1:**
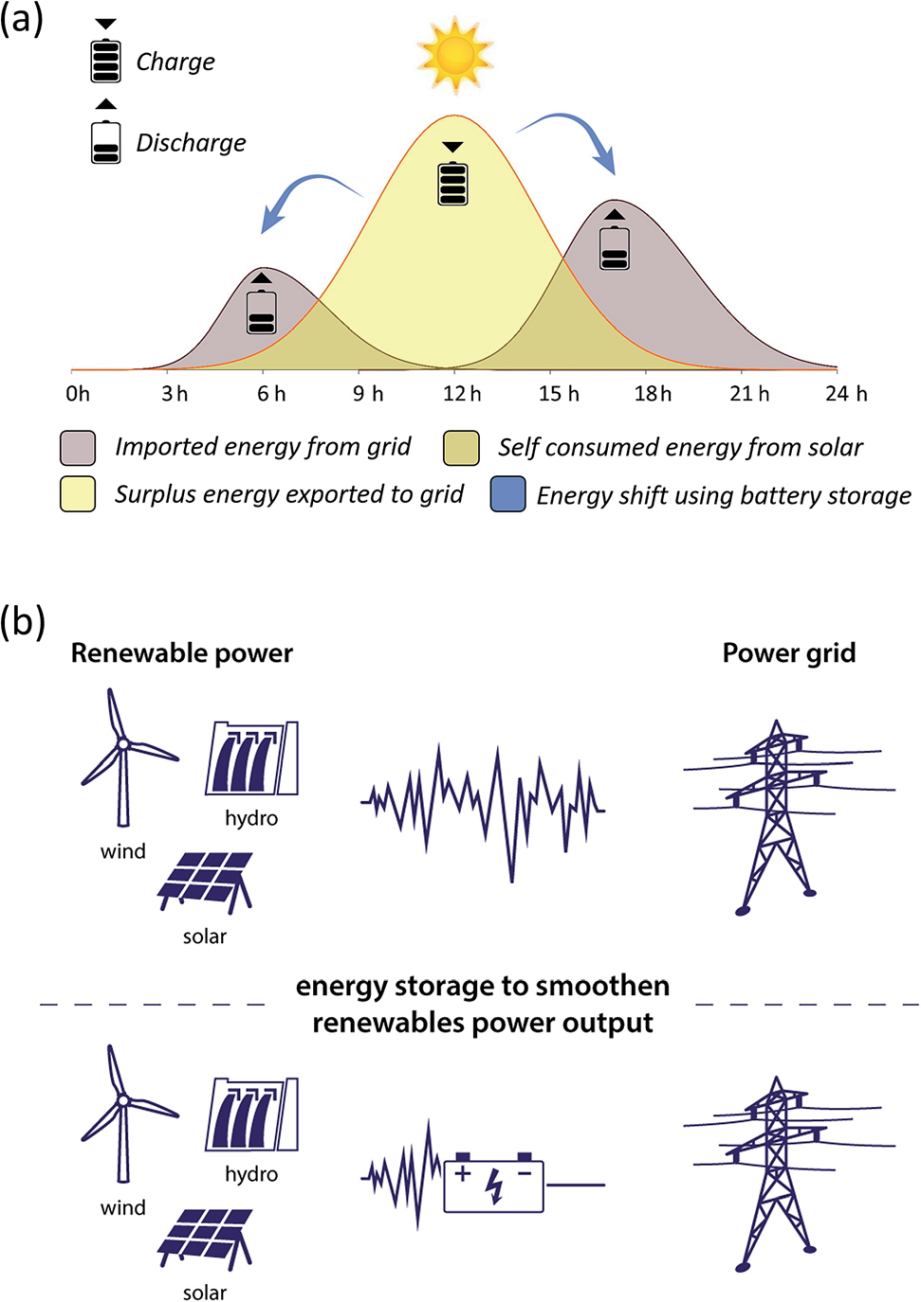
(a) Energy shift from solar power using battery storage. (b) Energy storage provides stored electricity to the grid and stable power output from renewable sources smoothening.

BESS are employed as a power or an energy configuration, depending on their intended application. In an energy configuration, the batteries introduce a steady amount of power into the grid so as to store excess renewable production for later periods of higher demand. Examples include energy supply shift process, peak management, back-up and energy time-shifting (Fig. 1(a)) and dispatch power over a period of many hours (energy trading or arbitrage). In a power configuration, the batteries inject a hefty amount of power into the grid during short intervals, which is achieved through the use of a high inverter-to-battery ratio. Both are deployed at the centralized solar and wind power production sites to smooth variable generation output (*i.e.* load levelling, voltage regulation, frequency response and regulation and peak shaving) as it is fed into the grid. These applications are distinguished from regulation because they occur on the renewable energy production side, storing energy directly generated from the specific renewable energy resources such as solar, wind and hydro (Fig. 1(b)). By contrast, battery storage regulation services operate at the grid level.

LIB technology is currently the most cost-effective solution for fast-response applications like frequency regulation and response as well as short-term spinning reserve applications (between 30 minutes and 3 h).^10^ As such, it holds the lion's share (>60%) of the total current utility-scale grid connected BESS market followed by sodium based batteries with 19%.^11^ Yet, over the last years there have been several reported fire incidents at LIB sites^12,13^ raising questions on its safety. On top of the above, evidence of a growing market demand for more flexible, mid to long duration applications (>3 h) is strong and imminent, enabling researchers to look for different and safer battery chemistries. Long duration storage technologies have the advantage of being more versatile in capturing a wider range of use cases and potential revenue streams.

**Fig. 2:**
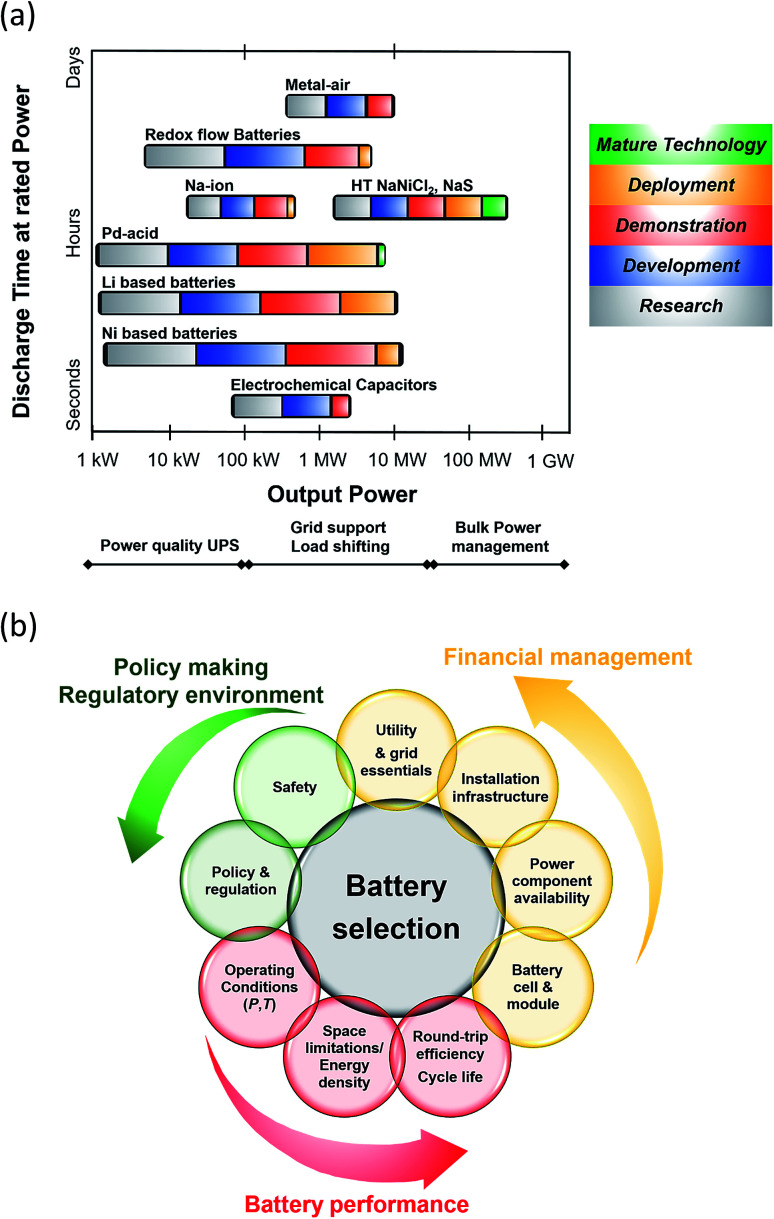
(a) Current status of Battery Energy Storage Technologies (BESS). Data taken from ref. 17 and 18. Metal air batteries: Zn–air and Li–air; RFB: Zn–Ir, Zn–Br_2_, Zn–NiO, Ir–Cr, VRFB. Pb–acid batteries: advanced and valve-regulated. Li based batteries: LTO, LCO, LFP, NMC, NCA and Li-polymer. Ni based batteries: Ni–Cd, Ni–Fe. (b) Criteria for BESS selection.

From a technological perspective, battery storage has matured enough to be considered a reliable and robust supplier of energy (Fig. 2(a)). Bain & Company estimates that by 2025, large scale battery storage could be cost competitive with peaking plants and that is based only on cost, without any of the added value companies and utilities the corporations are expected to generate from storage.^14^ In addition, Lux Research forecasts a $8.5 billion energy storage market by 2025 with transport applications covering 85% share of the revenues ($7.4 billion) while stationary applications will earn $1.3 billion.^15^

A breakdown of the key technical, financial and policy-making features surrounding BESS is outlined in Fig. 2(b). It is imperative that all these considerations are met in order for the BESS to operate in a sustainable, regulatory and cost-effective manner. Technical issues are linked to the life, performance and topography of the battery whilst financial considerations include power component availability and cost as well as maintained costs and risks related to manufacturers and vendors. Policy and regulatory aspects along with the appropriate safety measures are correlated to each specific battery technology and application.

## 2 Sodium battery technology

**Fig. 3:**
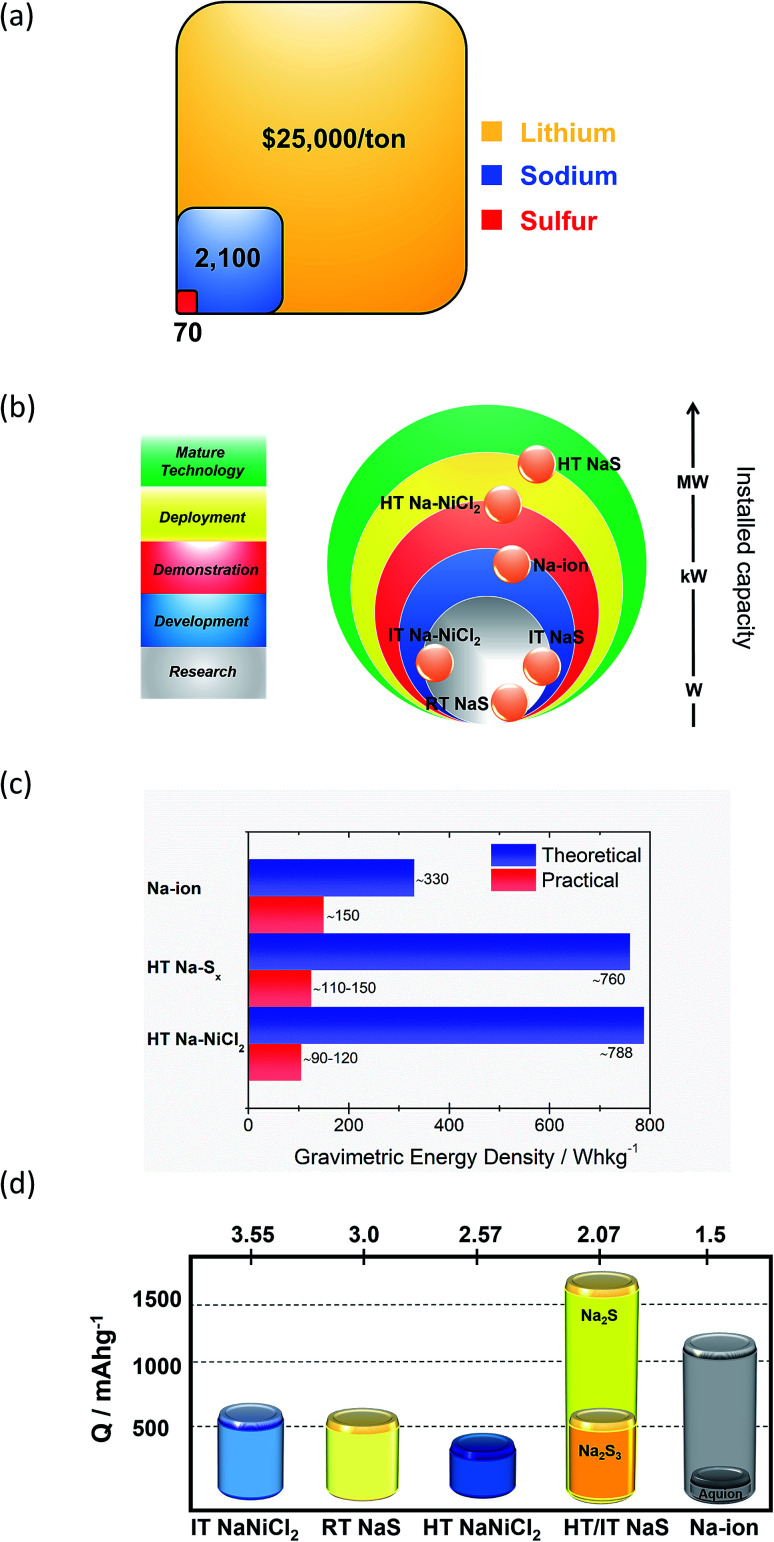
Na-based electrochemical energy storage systems. (a) Price breakdown of raw materials of the battery and comparison with lithium. (b) Current development status of the main Na-based technologies. Data taken from ref. 17. (c) Gravimetric energy density. (d) Capacity of various Na-based storage systems. Data taken from ref. 20, 40–42 and 48–52. Values of *E*_cell_ are at a fully charged state. For the RT NaS, the theoretical capacity of the cell is the capacity calculated from the weight of sulfur in the cell assuming full conversion to Na_2_S_*x*_ (0 < *x* < 2). For the HT Na–NiCl_2_, a molar ratio of 1.8 between Ni/NaCl is assumed.

Technological maturity of the BESS determines its selection priority for any given energy storage application. These technologies are preferred due to their developed operational expertise, which drives down the cost of the given technology. The significantly higher cost of raw lithium *versus* sodium renders the last one a more attractive choice for use in BESS (Fig. 3(a)). Also, LIB typically contains 5–20% Co, which is another expensive and scarce metal.^16^ Two main types of matured sodium batteries based on the materials used for the positive electrode are identified, namely the HT NaS and ZEBRA systems, both of which use liquid metal electrodes.

The ZEBRA technology is currently developed and commercialized by SoNick® systems.^19^ NaS refers to a commercialized tubular HT NaS system commercialized from Tokyo Electric Power Company (TEPCO) and NGK Insulator Ltd in 2002. The technology though was initially developed from Ford Motor Company in the 1960's followed by NASA. The operating temperature of this battery is high compared to its peers such as Pd–acid, redox flow and LIB due to the fact that polysulfide melt solidifies below 280 °C and the ionic conductivity of the solid electrolyte is heavily compromised at lower temperatures too.

Currently over 300 deployed energy storage stations with ZEBRA and HT NaS technology are in operation worldwide^19^ and offer attractive, cost-competitive technology for large scale storage of electrical energy. MW power battery modules are common for these battery systems since large unit cells mitigate the economic considerations by achieving low unit costs.^20^ Therefore, as depicted in Fig. 3(b) their maturity level is far more progressed when compared to other Na based technologies. The driving force for the realization of RT and IT NaS is the projected energy density gain from the decrease of temperature along with improved safety.^21,22^ The same rationale was implemented in ZEBRA batteries along with the introduction of a planar cell configuration. SIB technology due to the notable number of new electrode materials investigated in the last decade is at a more progressed level than the RT and IT NaS and Ni–NiCl_2_ systems.^23,24^

HT NaS and ZEBRA exhibit a large theoretical gravimetric energy density due to the high solubility of their active compounds. For the NaS system, the sulfur cathode along with the sodium anode can deliver a theoretical energy density of 760 W h kg^−1^, that is two times higher than Pb-acid.^25,26^ Yet, the actual operational energy density lies between 180 and 220 W h kg^−1^ (1000–1200 W h per 5.5 kg unit NaS cell), arguably promoting a longer calendar life with stable performance.^27–29^ The NGK cells demonstrate energy and power densities of 367 W h L^−1^ and 36 W kg^−1^, respectively with the optimal temperature range engaged between 300 and 340 °C.^30^ For the ZEBRA system, power density lingers around 166 W h kg^−1^ (100 W h per 600 g unit Na–NiCl_2_ cell).

Capacity-wise, a complete discharge of elemental sulfur to sodium sulphide (NaS cell) involves a conversion reaction with two electrons per sulfur atom and could yield a theoretical capacity of 1672 mA h g^−1^ (Fig. 3(d)).^31^ However, the reversibility of the system is in peril when going to lower polysulfides (Na_2_S_*x*_, *x* < 3) due to their insoluble nature at the battery's operating temperature and therefore a more realistic capacity points to 558 mA h g^−1^ at the state of sodium trisulfide (Na_2_S_3_) formation during discharge.^32^ The RT NaS cell shows a similar theoretical capacity (560 mA h g^−1^).^33^ Regarding the ZEBRA battery, which is the most prominent metal halide battery (Na-MH), the HT cell exhibits a lower capacity (305 mA h g^−1^) than NaS.^20^

Other than the ZEBRA cell, Na-MH batteries that have been reported in the literature include Na–FeCl_2_, Na–SbCl_3_ and Na–ZnCl_2_ systems with limited deployment success though, due to fast material degradation at high operating temperature and high manufacturing cost.^34,35^ To realise competitive molten Na-MH batteries in terms of safety, energy efficiency and density, the operating temperature has be set between 100–150 °C. In order to get to this temperature range (a) solid electrolytes with high ionic conductivity, good mechanical strength should be devised and (b) novel molten-salt electrolytes that possess a lower melting point than NaAlCl_4_ should be realized.^36^ An example of a novel molten salt battery working at IT is the one developed by Sumitomo Electric Lightwave and Kyoto University that uses a nickel cathode and glassy carbon anode. This cell is composed of non-flammable materials and delivers reasonable gravimetric capacities (*i.e.* 580 mA h g^−1^ assuming a potential of 2.57 V) along with closer cell packing at 90 °C.^37^ Former battery concepts based on liquid sodium include the NaBi cell with NaF–NaCl–NaI (15 : 32 : 53 mol%), the Na–Sn battery incorporated with a NaCl–NaI electrolyte (Na|NaCl–NaI|Sn) and the Na–Hg cell. However, they did not manage to go past the demonstration stage due to their high operating temperature (>580 °C) and environmental issues (toxicity of mercury).^38,39^ SIB currently utilizes a fraction of its theoretical capacity (*viz.* 30 mA h g^−1^*vs.* 1160 mA h g^−1^)^39,47^ while the Na–O_2_ cell despite having a theoretical specific energy of 1600 W h kg^−1^, it is hindered by the irreversible formation of sodium peroxide and superoxide.^43^

Clearly, HT Na-based systems are economically and technologically viable large scale storage solutions that exhibit manageable sustainability issues. Unlike other generating equipment, they do not generate CO_2_ or other gases and have a low environmental load due to their sealed structure. An increasing number of patents during the last years has been identified^44^ while also a tenfold increase of the installed rated capacity, reaching currently 4 GW h, outperforming any other commercialized Na-based system (HT NaNiCl_2_ = 7 MW h).^19^ Of late, the HT NaS technology is deployed in new parts of the world (Middle East, Africa, Europe and China) enhancing its technology penetration. Taking into account the increasing demand for energy storage, future improvements on the decrease of temperature and accessing the lower polysulfide spectrum in a reversible manner could render the NaS electrochemical energy storage systems price competitive with pumped hydro and compressed air storage. On a final note, it should be noted that a plethora of upcoming energy storage systems (*i.e.* solid oxide electrolyzers, fuel cells, redox flow cells) can also leverage the use of this existing technology.

## 3 Redox active components of the NaS battery

The energy released from the reaction of sulfur with sodium is the cornerstone of the NaS battery technology. Elemental sulfur contains a number of allotropes and several molecular structures with the most stable being the orthorhombic α-S crystal at ambient temperature and pressure.^45^ α-S is composed of puckered S_8_ rings organizing in an orthorhombic cell.^46^ In its elemental form, it shows as an odorless, non-metallic, pale-yellow solid. Sulfur is one of the chemical industry's most important raw materials, utilized as the derivative in numerous industrial processes including rubber processing, cosmetics and pharmaceuticals. The growth of the global sulfur industry is currently at a nominal rate of ∼2.5% and projected to reach 75 million metric tons by 2020.^47^ Measured in $kA h^−1^, sulfur has 0.15, zinc 3.66, graphite 32.27, and LCO 292.14 rendering it a financially sustainable chemical fuel material for energy storage.^2^

Sodium is a soft, lightweight metal that advertises itself as a robust, cheap and favourable chemical fuel. It is the 6^th^ most abundant metallic element in the earth's crust and 4^th^ in the ocean. More precisely, sodium content is 28 400 mg kg^−1^ and 1000 mg L^−1^ in the earth's crust and water (1.08% of ocean mass) in the form of numerous minerals such as rock salt and sodality.

It is available in numerous parts of the world namely Chile, China, Australia and Argentina, which hold 99% of its resources.^53^ Currently, metallic sodium is priced at 2100 USD ton^−1^ in quantity.^54^

Sodium provides an electrochemical reduction potential of −2.71 V *vs.* standard hydrogen electrode (SHE). When coupled as an anode with an appropriate cathode material it is capable of giving a cell voltage greater than 2.5 V. From its atomic mass (23 g mol^−1^), a theoretical capacity density of 1.16 A h g^−1^ can be realized. Sulfur possesses a theoretical capacity of 1.67 A h g^−1^ and has a formal potential of −0.407 V *vs.* SHE (S + 2e^−^ ⇌ S_2_^−^). Overall, the combination of high voltage and relatively low mass promotes both sodium and sulfur to be employed as electroactive compounds in electrochemical energy storage systems for obtaining high specific energy, especially at intermediate and high temperatures (100–350 °C).

## 4 Types of NaS battery

The types of NaS battery can be categorized by their operating temperatures. The major components of the HT (300–350 °C) and IT (150–200 °C) NaS cells are the solid ceramic electrolyte of β′′-alumina (BASE), the electrodes of sodium and sulfur in liquid state (*T*_mNa_ = 98 °C and *T*_mS_ = 118 °C) and a container. The cell configuration can be represented as Na(l)|β′′-Al_2_O_3_|Na_2_S_*x*_(l) + S(l)|C, where C stands for carbonaceous material that is commonly used as current collector and β′′-Al_2_O_3_ (beta-alumina) is the solid electrolyte. In the case of the RT NaS system (25–60 °C), metallic sodium is used instead of molten sodium and therefore BASE can be replaced by inexpensive glass fiber, the latter being a sound economic incentive for utilizing RT technology. The following sections discuss in detail the chemistry, current status and challenges of each of the major components of these battery systems.

### 4.1 HT NaS battery

The operation of the cell involves the formation of polysulfides, *viz.* chemical compounds containing chains of sulfur atoms that are complexed with molten sodium forming sodium polysulfides. Molten sulfur being a covalent bond species is usually impregnated into porous carbon-based current collectors placed in-between the BASE and the cell container, to provide sufficient electronic conduction to carry out the electrochemical reactions.

**Fig. 4:**
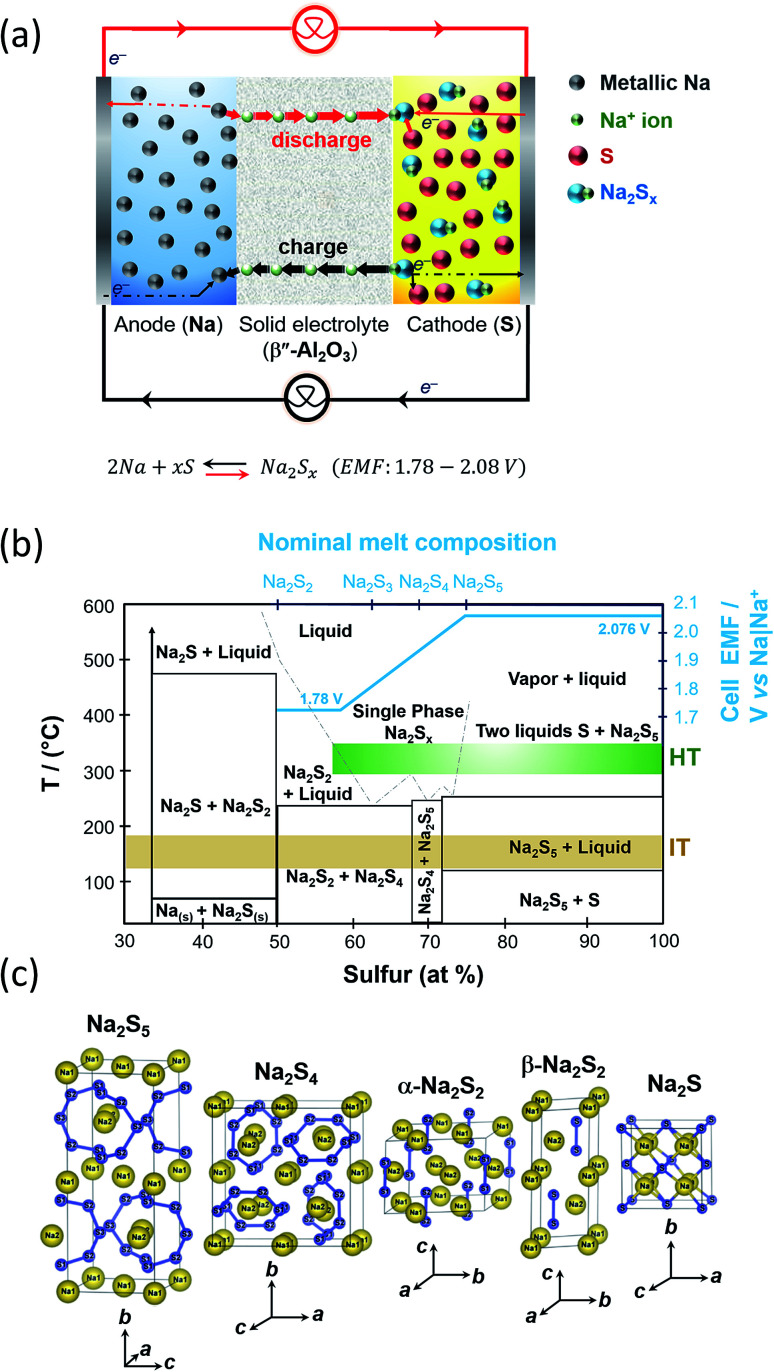
(a) Diagram depicting the operation of the HT/IT NaS battery. (b) Phase diagram of the HT/IT NaS system for the commercialized HT system. Temperature *versus* atomic percentage of sulfur. Cell EMF (blue line) at 350 °C. The diagram is adapted/reproduced from ref. 55 with permission from the Electrochemical Society. (c) Crystal structures of NaS_*x*_ materials showing the S–S chain structures of each polysulfide. Diagram is adapted/reproduced from ref. 46 with permission from the *Journal of the Physical Society of Japan*.

During discharge, sodium ions travel through the BASE to the sulfur cathode and electrons flow in the external circuit of the battery. Then, they react with sulfur to produce sodium polysulfide intermediates as depicted in Fig. 4(a). Electrons are transported to the reaction sites where the carbon fibers are in contact with the BASE. During the subsequent reversible charge process, sodium polysulfides release the positive sodium ions through the electrolyte to recombine as elemental sodium at the anode polysulfides.

Molten sodium make intimate contact with BASE and gives rise to negligible ohmic polarization caused by contact resistance at the Na|β′′-Al_2_O_3_ interface. The operational potential window 2.08 and 1.78 V, being dependent on temperature and melt compositions as depicted in the Na|Na_2_S_*x*_ phase diagram first devised by Gupta and Tischer and perfected later on through theoretical calculations and further experimental results.^56,57^

At the cell's operating temperature, there are two phase regions where the melt is completely ionized containing sodium and sodium polysulfide ions solely.^58^ When the starting anodic and cathodic reactants are in their pure elemental forms the cell electromotive force in the two phase region is 2.076 V (Fig. 4(b)). At compositions of *x* > 5, that is the sulfur rich state, the melt consists of two liquid phases that solidify at 253 °C (sodium pentasulfide, Na_2_S_5_) and 115 °C (β-S_8_), respectively.^59^ Any capacity loss evidenced in this system stems from inadequate charging at the two-phase zone.^49^ During the early stage of discharge, sulfur content ranges from 78 to 100 wt%. The initial stage of discharge is described by eqn (1).

1 2Na + 5S → Na_2_S_5_*E*_300 °C_ = 2.076 V

Since there are two components and two phases, from the Gibbs rule we can deduce that there are only two degrees of freedom, namely temperature and pressure, whilst pressure is invariant. The discharge begins adjacent to the Na|β′′-Al_2_O_3_ and the two phase zone gradually extends through the catholyte to the current collector. As this zone thickens, the sodium content increases upon complete filling of the cathode with Na_2_S_5_. Upon prolonged discharge, all sulfur combines with sodium to form a polysulfide in the shape of sodium tetrasulfide (Na_2_S_4_) as illustrated in eqn (2), which consecutively results in the appearance of a third degree of freedom that being the potential. The latter in turn prompts the cell voltage to fall progressively. The melting point of Na_2_S_4_ lies at 290 °C whereas polysulfide melts with compositions of Na_2_S_*x*_ (3 < *x* < 5) solidify at lower temperatures (<290 °C).

2 2Na + 4Na_2_S_5_ → 5Na_2_S_4_*E*_300 °C_ = 1.970 V

The reaction proceeds until an overall melt composition equivalent to *x* = 3 is achieved, according to eqn (3). Further discharge to sodium trisulfide (Na_2_S_3_, *T*_m_ = 235 °C) leads to the EMF decreasing to 1.78 V, where it can remain as long as the liquid phase is present.^56^

3 2Na + 3Na_2_S_4_ → 4Na_2_S_3_*E*_300 °C_ = 1.74–1.81 V

Density functional theory (DFT) calculations predict Na_2_S_3_ to be of *C*_2v_ symmetry with the coordination of the metal by the terminal sulfur atoms of the S_3_ unit only.^46,60^ The enthalpy of formation of Na_2_S_3_ (−0.85 eV per atom) is greater than the one of Na_2_S_4_ and Na_2_S_5_, respectively (−0.80 and −0.70 eV per atom). Beyond this point, sodium disulfide (Na_2_S_2_, *T*_m_ = 475 °C) is formed as corroborated by density functional calculations and the phase diagram of Fig. 4(b). The Gibbs free energy of formation from Na_2_S_2_ is negative^60^ and precipitates in the electrolyte being electronically non-conductive, hindering battery recharge. Hence, in order to ensure continued operation, the cell should be discharged up to Na_2_S_3_. Fig. 4(c) shows the crystal structure of sodium disulphide, which consists of S_2_ dimers, namely α-Na_2_S_2_ and β-Na_2_S_2_. Theoretically, full discharge is terminated when the voltage reaches 1.78 V. Sulfur reacts with sodium ions to form sodium polysulfides according to eqn (4):

4 2Na + (*x* − 1)Na_2_S + 2e^−^ → *x*Na_2_S_(*x*−1)_

*x* varies from 2 to 6 depending on the overall composition of the melt. In the higher polysulfides, the metal atoms interact not only with the terminal atoms of the sulfur chain but also with adjacent atoms resulting in cluster-like structures of low symmetry and in branching of the polysulfide anion (Fig. 4(c)).^60^

In practical HT NaS cells, concentration gradients of sulfide anions and sodium cations are created in the melt by the migration of the sodium and sulfide ions. The extent of these concentration gradients depends on the magnitude of transference numbers of the sodium ion and to a lesser extent of the sulfur anion. It is postulated that the principal current carrying species are the sodium ions, owing to (a) their larger relative ion size; 1.02 Å *vs.* 0.37 Å for sulfur and (b) the diffusivity of the individual ions that tend to counteract the movement of species by migration.^61^

#### 4.1.1 BASE

The essential requirements for a reliable solid electrolyte involve a high sodium ion conductivity with zero electronic conduction, chemical corrosion resistance to sulfur and polysulfides, impermeability and adequate thermal and mechanical strength. The beta alumina type of crystal structure is ideally suited for rapid ionic transport in the conduction planes, having numerous defect sites for the mobile ions linked with networks and allowing easy access. The properties of beta alumina resemble those of more conventional ceramics, so that electrolyte tubes with acceptable thermal and mechanical properties can be constructed in large numbers.^49^

**Fig. 5:**
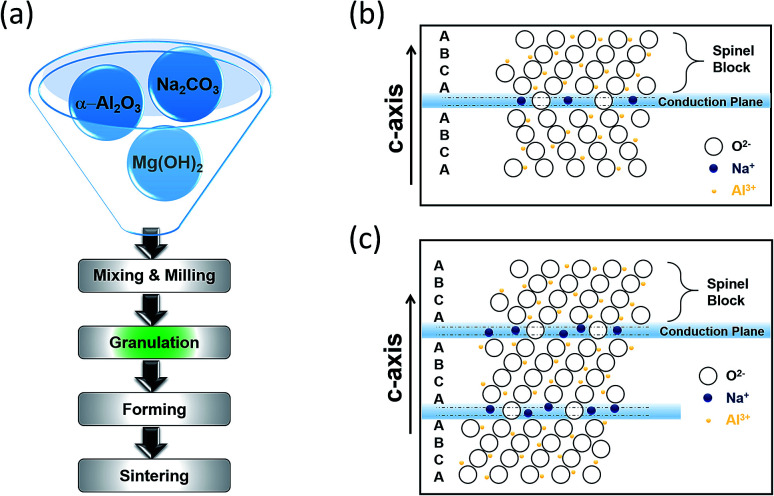
(a) Conventional tube (β′′-Al_2_O_3_) production process. Crystal structure of (b) β-Al_2_O_3_ and (c) β′′-Al_2_O_3_. The diagram is adapted/reproduced from ref. 67 with permission from *Elsevier*.

Borate glass and beta alumina are the building blocks of the BASE. The resistivity of the glass (∼1000 ohm cm^−1^) is higher than that of beta alumina by a factor of 10^2^ leading to the formation of extremely thin walled capillary tubes. The beta alumina powders can be synthesized *via* several techniques including sol–gel, solid-state reaction, co-precipitation and the spray-freeze/freeze-drying.^62,63^ The traditional synthesis of beta alumina involves a HT solid state reaction of α-Al_2_O_3_ with Na_2_O in the presence of Li_2_O and/or MgO, the last two acting as stabilizers. The raw materials are initially mixed and milled followed by granulation where the formation of a green product is evidenced. Whilst the tube is fired up, the transition from α-alumina to β-alumina takes place. It is imperative that the alumina surface is protected by materials that are impervious to water and in the same time preserve the sodium ion conductivity. Firing conditions such as the sintering step of Fig. 5(a) must be carefully managed to obtain crystal grain sizes and grain size distributions that provide an optimal combination of strength and conductivity.

Beta alumina has the general formula of Na_1+*x*_Mg_*x*_Al_11−*x*_O_17_ (*x* ≈ 0.67). Sodium beta alumina is a 2D conductor, made of alkaline powder that is sensitive to moisture and has the general structure Na_2_O·*x*Al_2_O_3_ and an activation energy of 0.16 eV ± 0.01 eV. Depending on *x*, two distinct crystal structures are formed, beta alumina (β-Al_2_O_3_, *x* = 8–11) and beta′′ alumina (β′′-Al_2_O_3_, *x* = 5–7).^64^ These structures enable sodium ions to move freely in conduction planes that have a high proportion of defect sites liked by easily accessible networks. Fig. 5(b) and (c) illustrate the β and β′′-Al_2_O_3_ unit cells on (112̄0), showing a stacking sequence. The basic unit consists of spinel blocks and conduction planes. The spinel block has a four layer ABCA stack of oxygen ions encircled with aluminum ions whereas the conduction planes or slabs contain fewer oxygen atoms giving more room for sodium cations. One full unit cell requires three sets of this pairing. The bridging oxygen atoms in the loose layers provide spacing, which in turn allows the interstitial migration of sodium ions in the conduction plane. The difference between the materials stems from their chemical stoichiometry, sodium ion conductivity and stacking sequence of O^2+^ across the conduction layer. The unit cell of β′′-Al_2_O_3_, stacked according to a threefold screw axis containing no mirror plane and having a rhombohedral structure is 50% larger than that of β-Al_2_O_3_, giving an enhanced sodium ionic conductivity, suitable for battery applications as in the case of the HT NaS cell.^65,66^

The ionic conductivity of the β′′-Al_2_O_3_ at 350 °C is similar to that of an aqueous electrolyte in (non)aqueous batteries when at the same time the material remains virtually an electronic insulator. At 25 °C, single crystals in the plane direction reach 0.1 S cm^−1^ whereas commercial β′′-Al_2_O_3_ reach 0.002 S cm^−1^. At 300 °C, the conductivity of a single β′′-Al_2_O_3_ crystal lingers at 1 S cm^−1^ compared to 0.3 S cm^−1^ for polycrystalline β′′-Al_2_O_3_ at the same temperature.^68^ The higher conductivity of single crystals is attributed to the absence of grain boundary effects and anisotropic ion conduction in the β and β′′-Al_2_O_3_ crystals.^29^ The diffusion of ions through the β′′-Al_2_O_3_ is a thermally activated process. At 300 °C, the surface of the electrolyte in the cell is partially wetted and it becomes fully wetted at 350 °C.^68,69^ Still, impurities like calcium (Ca) and lithium oxide (Li_2_O) that are formed during cell operation can impede sodium wettability during long-term cycling by forming a blocking calcium oxide (CaO) film between sodium and the BASE.^49,70^

Overall, the BASE conveniently and effectively allows sodium ion transport, rendering the HT NaS a competitively energy storage system. It is largely dependent on temperature and can be somewhat prone to formation of blocking layers upon long term cycling and exposure to air. Apart from the traditional and energy intensive method of manufacturing BASE, a number of different methods have been reported including tape casting, sol–gel, co-precipitation, hydrothermal growth and nanopowder processing.^62,66,71–74^

#### 4.1.2 The sulfur electrode

The sulfur electrode is responsible for the rechargeability of the cell. The electrochemical potential, stoichiometry, and composition of the polysulfide melt varies periodically during cell operation, which in turn alters the solubility, wetting properties, chemical reactivity and viscosity of the reaction products. In the liquid phase, polysulfide anions of different chain lengths are in equilibrium with each other as corroborated by *ab initio* computational calculations that show that in all polysulfides, sulfur exists in the form of unbranched S_*n*_^2−^ chains.^75,76^ This equilibration is often described by the following equation:

5 2S^2−^_*x*_ → S^2−^_*y*_ + S^2−^_*z*_ (2*x* = *y* + *z* and *y* > *z*)

Elemental sulfur between 300–350 °C is a viscous liquid with a measurable vapour pressure (bp: 444 °C) that is reduced to polysulfide ions on discharge and reformed on charge. The initial cell discharge involves the formation of Na_2_S_5_, having a density of 1.86 g cm^−3^, which is higher than that of sulfur at 350 °C (1.66 g cm^−3^), indicating that the latter forms the upper phase in the two phase system of Fig. 4(b).^49^ The density of Na_2_S_3_ at 350 °C is similar to that of Na_2_S_5_, equal to 1.87 g cm^−3^.^49^ At 300 °C, the densities of the polysulfides do not differ markedly, 1.89 and 1.90 g cm^−3^ for Na_2_S_5_ and Na_2_S_3_, respectively, yet the surface tension has been reported to increase by a factor of three.^77^ The conductivity of sulfur at 300 °C is 10^−8^ Ω cm^−1^, considerably higher than in 25 °C, *viz.* 5 × 10^−30^ Ω cm^−1^, while that of for Na_2_S_5_ and Na_2_S_3_ are of the order 0.18 and 0.41 Ω cm^−1^, respectively.^77,78^ Diffusion coefficients (*D*) on polysulfide melts at the single phase region (between Na_2_S_3_ and Na_2_S_5_) at 350 °C linger between 1 × 10^−5^ cm^2^ and 6 × 10^−7^ cm^2^ s^−1^ for Na_2_S_5_. In the case of Na_2_S_4_, *D* was reported equal to *ca.* 2 × 10^−7^ cm^2^ s^−1^. For Na_2_S_3_, the *D* values were of the order of *ca.* 9 × 10^−6^ cm^2^ s^−1^.^79,80^ The reversible charge transfer reaction represented by eqn (6) can partially describe the reaction condition on the sulfur electrode:^65^

6 (*x* − 1)S_*x*_ + 2e^−^ ↔ *x*S_(*x*−1)_

High currents can be realized from the sulfur electrode under the provision of high electrode surface area and solubility of sulfur in the polysulfide at the electrode/melt interface.^81^ The highly corrosive environment though constitutes a major issue and limits the selection for current collectors and containers. Molybdenum (Mo), chromium (Cr), aluminum (Al) and stainless steel (SS) have been tested as current collectors with moderate success due to economic and practical reasons.^49^ A more suitable solution entails the introduction of cost-effective corrosion resistant alloys like stainless steel and perovskites such as La_0.8_Sr_0.2_Co_0.3_Fe_0.7_O_3−*δ*_ (LSCF) that was found to retard the corrosion process of the Na_2_S_4_ melt significantly.^62,82^

Several carbon-based materials have also been explored as an efficient hosts for sulfur due to their relative high conductivity, ease of tailoring their structure, chemical stability, large surface area and compliance with physicochemical characterization tools. The most common cathodic collectors include (modified) graphite felts and cloths with oriented fibers (radially, perpendicularly or crosswise) or needle-punched glass fibers (of ∼10 μm diameter), microporous carbon and graphite powders.^83,84^ Normally, the collector is wrapped with stainless steel or tungsten wire around.^85^ Examples of carbon felt modification involve the introduction of an inorganic–organic hybrid sol layer that enhanced the charge acceptance and allowed the cell to operate at current densities as high as 100 mA cm^−2^ (ref. 86) as well as sulfur dioxide and α-Al_2_O_3_ coatings that improved the electrochemical behavior of the cell.^49,63^ Microporous carbon can initiate fast electron transfer for high capacity as it offers a larger active surface area to carry the electrochemical reactions during the operation of the battery.

**Fig. 6:**
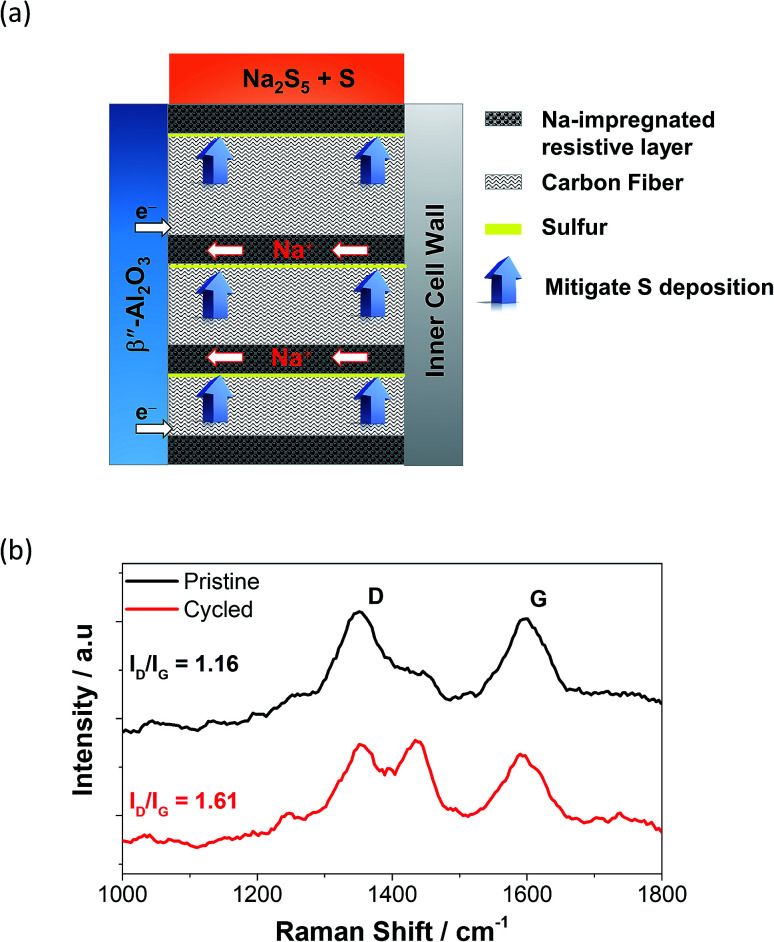
(a) Structure of a perpendicular cathode on a tubular HT NaS cell. This figure has been adapted/reproduced from ref. 87 with permission from *Elsevier*. (b) Raman spectroscopy of carbon cloth current collector on a NaS cell containing 2 M Na_2_S_5_ and TEGDME. Cycled carbon cloth shows the use of the cell for 50 charge–discharge cycles at 160 °C and a current density of 2 mA cm^−2^. D band arises from the disordered structure of graphene. G band arises from the stretching of the C–C bond in the carbon cloth. The appearance of an extra peak signifies different defect densities; taken from ref. 93.

In order to combat the poor wettability of sulfur on the carbon felt, the implementation of different cathode structures has been reported.^87^ Layered structures containing carbon fibers accompanied by a highly resistive layer are placed perpendicularly to the BASE acting as a passage way for the sodium ions to reach the anode and also as a sulfur deposition suppressant are displayed in Fig. 6(a). Similarly, the introduction of round to clover-leaf shaped BASE in a ZEBRA cell managed to (a) enhance the active electrochemical area and (b) minimize the thickness of the cathode, leading to greater power densities.^88^ Other reported strategies involve the introduction of an overlayer (*e.g.* Al/Si) or casting on the BASE to construct electronically-conductive matrixes where sodium polysulfides can freely migrate, whilst in the same time isolating sulfur from the carbon fibers drastically improving the internal resistance of the cell is essential.^86,89^

Raman spectroscopy is a helpful tool that can accurately detect the presence or absence of sulfur molecules on the current collector on top of the detailed bonding structure of the carbon material itself.^21^ The carbonaceous materials reveal the characteristic G (sp^2^ hybridized) and D (sp^3^ hybridized) modes at 1360 and 1592 cm^−1^, respectively.^90^ The *I*_D_/*I*_G_ ratio is a reliable indicator of the extent of structural defect of the carbon based material with regards to usage. Fig. 6(b) shows that the *I*_D_/*I*_G_ ratio of the carbon cloth shifted from 1.16 from 1.61, indicating a substantial level of defect on its structure upon repetitive galvanostatic cycling.^90,91^

In order to enhance polysulfide solubility and inhibit non-desirable discharge products such as sulfur deposition on the BASE, the employment of various additives has been suggested, namely tetracyanoethylene (C_6_N_4_),^49^ selenium (Se, 1 mol%),^92^ silicon dioxide (SiO_2_)^63^ metal sulfides like FeS_2_, NiS_2_, CoS_2_,^93^ carbondisulfide (CS_2_)^94^ carbon suspensions,^91^ and tetracyanoethylene (TCNE).^95^ For the Na-MH technology additives on the NaAIX_4_ electrolyte such as thionyl chloride (SOCl_2_), sulfur dioxide (SO_2_) and dimethyl sulfoxide (DMSO)^44^ have been successfully employed. The additives seek to stabilize the long chain polysulfides during discharge while in the same time improving sulfur solubility.

To sum up, the formidable material requirements for the sulfur electrode encompass good corrosion resistance, high conductivity, good wettability against the polysulfides formed during the cell operation and low weight and cost.

#### 4.1.3 The sodium electrode

The sodium electrode is as important as sulfur for the robust operation of the HT NaS cell. The solubility of liquid sodium in molten salts ranges between 1.6 and 3 mol% and its surface tension is equal to 200 mN m^−1^.^96,97^ The high reactivity of the molten sodium can afford high current densities of the order of 40 to 300 A cm^−2^, rendering it the limiting element of the cell performance.^49^ Sodium serves a dual purpose, that of a reactant and a current collector. When consumed during discharge of the cell, a volume decrease occurs. At this stage, it is paramount to keep sodium in contact with the active area of the BASE, thereby minimizing the anode resistance. The three most prevailing ways to achieve this involve (i) feeding sodium by gravity from a top reservoir (ii) wicking sodium to the BASE surface and (iii) forcing sodium from a reservoir by gas pressure.^68^ The interfacial polarization of the Na|β′′-Al_2_O_3_ appears to be dependent on the ceramic composition and can abruptly increase in a non-asymmetric manner. This behavior stems from the formation of sodium oxide film or from impurities arising from the ceramic electrolyte.^49,88^ Typical ways to diminish the interfacial polarization implicate renewing the sodium electrode, treatment of the ceramic surface to improve wetting and introduction of oxygen getters such titanium and aluminium into liquid sodium.

In order to devise a sodium electrode and taking into account its reactive nature and high electrical conductivity (*ca.* 2.1 × 10^7^ S m^−1^), the electrolyte separator must be thinner than the equivalent beta alumina membrane in order to keep the resistance low. It has to be mentioned that the sodium ion intermediate size (2.27 Å) offers no change in its conductivity up to pressures of 20 kbar. The sodium electrode can be realized by using glass in the form of hollow fibers with walls of certain distance, typically 10 mm.^49^ Once the issue of the interfacial polarization is dealt with, the liquid sodium metal can behave as an ideal reversible electrode.

#### 4.1.4 Cell considerations

##### 4.1.4.1 Cell design

A great deal of research has been done on devising the optimum design of this BESS. Tubular and planar cells are the prevailing designs each bearing its advantages and limitations. They exhibit different bulk transport mechanisms of their active species to the reaction front that is formed on the outside of the BASE.^84^

**Fig. 7:**
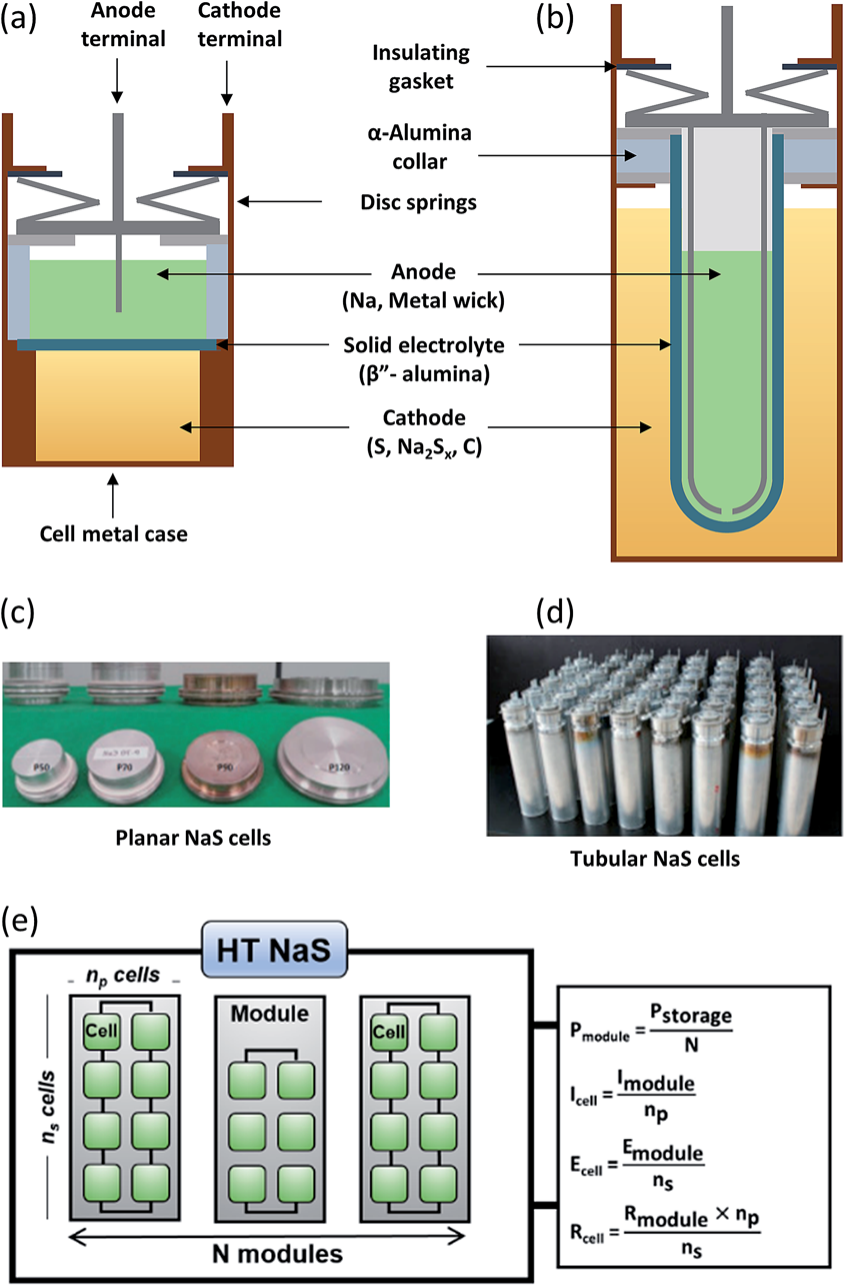
Components of (a) planar and (b) tubular NaS cells. Most parts of the battery are fabricated from inexpensive materials while the majority of the overall weight of the battery materials (99%) can be recycled (steel, copper, and aluminum). This figure has been adapted/reproduced from ref. 20 with permission from *Elsevier*. Optical pictures of planar (c) and tubular (d) NaS cells. This figure has been adapted/reproduced from ref. 100 with permission from *Elsevier* (e) NaS assembly of modules and cells with the respective equations describing its parameters (*E*: potential, *P*: power, *I*: current, *R*: resistance).

Planar cells (Fig. 7(a)) are widely used for fundamental studies on the battery chemistry such as wetting characteristics of molten sodium on the BASE,^98^*in situ* monitoring of polysulfides^28^ as well as testing of novel cathodes.^99^ They offer several distinct advantages including efficient stacking, direct inter-cell connection without any external connectors, better stability due to cathode geometry and larger active area of BASE per unit weight of the cell.^100^ Non-glass sealings can also be implemented here due to the lesser amount of joints and cell architecture. An example of such a compliant polymer sealing for a planar Na–NiCl_2_ battery is a fluorinated ethylene propylene (FEP)–polyvinylidene fluoride (PVDF) seal that could run at 190 °C for 1000 cycles with high capacity retention and no signs of degradation.^101^

A direct comparison study between planar and tubular HT NaS cells (Fig. 7(c) and (d)) revealed that the deviation from the ideal open circuit voltage (especially in the discharge process) was much smaller for the case of the planar cell, which can lead to a relatively more stable and high power operation by reducing the power loss compared with the conventional tubular cells.^84^ Despite these advantages, no commercial up-to date planar cells have been devised for practical operations due to the thermo-mechanical fracture issue in the cell joint or BASE area.

Commercial HT NaS cells implemented largely in load levelling and stationary applications are solely tubular based (Fig. 7(b) and (d)). No circulation is required for this system unlike other MH batteries (Zn–Cl_2_, Zn–Br). The prevailing design involves a cylinder-shaped metallic cell casing that also serves as the current collector, the sulfur electrode and a tubular BASE surrounding the internal metal container as well as the molten sodium.^83^ The cell is assembled by inserting the sulfur electrode in the cell casing, followed by the electrolyte tube containing the sodium electrode. An inert gas forces sodium onto the surface of the electrolyte. The electrolyte tube is closed off by a beta alumina hermetic seal together with an alpha alumina metal thermo-compression seal.^102,103^ Further refinements have emphasized on reducing sealing areas so as to avoid any large thermal mismatch during the thermal compression bonding (TCB) process.

The active materials (*i.e.* sodium and sulfur) account for approximately one third of the weight of the cell (32%) case whereas BASE accounts for 20%.^20^Fig. 7(b) displays all the main components of the cell including metal casing (30 wt%), disc springs, carbon (3 wt%), current collector (3 wt%) aluminum gaskets and sealing gaskets (12 wt%). As a figure of merit, a 1 MW battery module developed by NGK insulators weighs 86 tonnes^104^ (Fig. 7(d)). The emission of carbon dioxide from the whole manufacturing is of the order of ∼0.15 g per W h.

The HT NaS thermally insulated and vacuum-packed module, comprises of multiple unitary cells mechanically and electrically connected in an insulated enclosure as depicted in Fig. 7(e). A robust thermal management system for initial heat-up and waste heat control plus sound electrical networking and insulation in the form of double-walled stainless steel is enforced.^105^ For large utility applications, insulation takes the form of conventional fibers board or microporous materials that are cheap, flexible and can be utilized to provide energy for reinjecting lost heat.^106^ Its high spatial efficiency makes it an ideal candidate for realizing large individual cells or cell stacks. As an example, at the Buzen Substation in Japan, batteries with a storage capacity of 300 MW h are installed on a relatively narrow site measuring approximately 14 000 m^2^ comprising of 252 containers, currently being the largest battery system in the world.^31^ Modular fabrication can yield high power and energy capability, which can reduce the construction intervals.^107^

##### 4.1.4.2 Thermal management

The heat produced during charge is sufficient to sustain the operating temperature without heaters whereas the nominal temperature of the implemented system remains at 325 °C to successfully achieve combined operation and peak shaving.^108^ During discharge, the cell generates resistance and entropic heat leading to an increase in its temperature whereas during charge the amount of resistance heat generation is similar to that of entropic heat absorption.^109^ The temperature gradient between the inner and outer cells does not exceed 30 °C in order to maintain temperature uniformity and mitigate potential hazards to the external environment.^110^

The efficiency of the module is dependent on temperature uniformity inside the module, both in vertical and horizontal directions and the cooling and heat dissipation from the casing. The last two can be realized through the use of heat pipes, thermal shunts, latent heat storage, evaporative cooling and air/liquid heat exchange.^111^ A good electrical networking through the introduction of banks on each unit is crucial for maintaining battery performance in case of cell failure or aging as it can isolate the faulty cell. Temperature fluctuation can provoke TCB joint failures due to the distribution of thermomechanical stress during the booting and shutdown of the cell but can be mitigated by implementing a larger interfacial contact area.^100,112^ TCB is one of the most reliable solid state hermetic sealing methods implemented between heterogeneous components that comprise large sized HT NaS cells, including ceramic|ceramic, metal|metal, and metal|ceramic couples, electron-beam and gas tungsten arc welding.^113^

Each module is protected by a battery management system (BMS) that disconnects the battery from the load in case of anomalous behavior such as voltage values outside the predefined operating range.^114^ External heaters are intermittently used in case the system is idle or the operating temperature drops below 300 °C and are located at the bottom and the sides of the battery modules. The use of electric heating can improve temperature uniformity on the expense of module efficiency. A typical thermal loss from a 50 kW h battery is of the order of 200 W meaning that the battery can stand-off charge for up to 48 h.^103^ Yet, a more realistic thermal loss approximation for current HT NaS modules is of the order of 15% on a daily basis.^115^

#### 4.1.5 Safety

The high operating temperature range of the HT NaS system raises several safety concerns. Chemical hazards of elemental sodium and sulfur are rather substantial due to the high negative enthalpy of the reaction at 350 °C (Δ*H* = −420 kJ mol^−1^).^105^ Bulk sodium is stored in a corrosion resistant safety tube^103^ equipped with a small supply hole at its base that regulates the flow of sodium and also allows a minimum amount of sodium to maintain the electrochemical reaction. Sulfur is not as chemically reactive as sodium, yet it bears significant hazards associated with its use, namely flammability and release of toxic sulfur dioxide gas. It is clear from the phase diagram of Fig. 4(b) that the operating temperature of the HT NaS should not exceed 400 °C under any circumstance.

Water-free environment is a prerequisite due to the hygroscopic nature of polysulfides that can cause hydrolysis and form hydrogen sulfide and sulfur, which will in turn alter the melting point of the system and attack the BASE.^116–118^ To completely eradicate moisture penetration, inner and outer protection sheets are added for thermal insulation and fire resistance.^105,119^ The outer thermal container keeps the heat loss within acceptable limits, insulates the battery from external temperature extremes and forms the final protective barrier between any leaking reactants and the external physical environment. Thin double-walled evacuated enclosures are implemented to withstand water and salt spray, mechanical shock and vibration.^103,120^

Current cell designs utilize a chromium-containing layer as the primary corrosion barrier^103^ followed by stainless steel, the latter being useful in mitigating the radial stress concentration during the fluctuating operating temperature of this energy storage system.^30,112^ Further developments include the introduction of hypo-eutectic aluminum alloys (Al–Mg–*x*Si) that act as an inert metal for enhanced thermal compression bonding.^113^

Plausible scenarios of HT NaS cell failure entail cracking of the BASE due to non-uniform current distribution leading to high local current density degradation of the glass seal, corrosion of the container and breaching of the metal-to-ceramic seals. A rigorous safety testing protocol has been devised by NGK Insulators Ltd., involving self-extinguishing module and container design, container drop, fire resistant, flood, module drop and external short circuit. On top of the safety protocols, numerical simulations are utilized in order to limit the quantity of sodium available for reaction with sulfur in case of electrolyte fractures, restrict the flow of sodium to the site of the electrolyte fracture as well as protect the metal casing and current collector from corrosion by sodium polysulfides.^105,121^ In order to minimize the above, a “safety tube” is placed inside the BASE that limits the maximum temperature and pressure the cell can attain during operation.

Glass sealant should match the thermal expansion of the ceramics as well as the chemical stability of the component materials. It has been suggested that bismuth oxide (Bi_2_O_3_, 12–42 wt%) glass ceramic sealants can enhance the thermal shock resistance of the sealant and impede any spread of microcracks.^122,123^ In an array of cells such as in Fig. 7(e), the gaps between the cells are filled with engineering sand made of silicon dioxide to prevent fires. Through the use of a TCB the airtight and watertight sealing can withstand temperatures up to 650 °C.^49^

The integrity of the cell is monitored closely for all possible faults such as cell damage, material leaks, deformation of modules and fires. This is attested by the track record of this energy storage system with numerous deployed stationary applications in numerous countries, several successful decommissions and only one recorded incident at the Mitsubishi materials construction plant at Tsukuba, Japan in 2011.^108^ NGK post mortem analysis pinpointed that the possible cause of the fire was due to the expulsion of electrolyte from a single cell caused by a short circuit, triggering a cascading failure of multiple cells. The actions taken to remedy the situation on future cells entailed the addition of fuses between modular battery cells along with insulation and anti-fire boards between them.^124^

#### 4.1.6 Battery life cycle analysis

The primary material production energy for a kilogram of each material (j) labelled as PE_j_ is not significant for sulfur and sodium to a lesser extent, giving PE_j_ values of 5 and 150 MJ kg^−1^, respectively. For comparison purposes, the energy consumption of LMO is 35 MJ kg^−1^ and for LCO significantly higher, *viz.* 80 MJ kg^−1^.^125^ In addition, the HT NaS battery consumes three times as much energy per watt hour as the Pd–acid battery but is comparable with Ni–Cd and LIB batteries.^26^

The production of the BASE is energy intensive primarily due to the sintering process, leading to PE_j_ values greater than 500 MJ kg^−1^. Nonetheless, this verified ceramic component is automated^126^ meaning that there is room for improvement through high production yields and economies of scale. Solution based methods are preferred since they decrease the sintering temperature and produce more homogeneous powders.^63^ Lastly, on an environmentally friendly note, over 99% of the materials that make up the HT NaS battery can be recycled with sodium being handled as hazardous. There have been several successful decommissions of this system, namely the 48 MW h modules in Shizuoka and Ohito, Japan, and the 6 MW h in Lerwick, the Shetlands.^17^

#### 4.1.7 Current commercial status

A stable capacity for the HT NaS system can be obtained for 4500 cycles to 80% depth of discharge (DOD) at a discharge rate between 8–10 hours that translates to a calendar life of at least 15 years and a millisecond response for full charging and discharging operation.^20,107,113^ Typically, the state of charge (SOC) of the battery is confined between 20 and 95% so as to avoid parasitic and self-discharge reactions and contain the high electrical resistivity of the BASE. High coulombic and mean round trip efficiencies can be realized, *viz.* >98% and 82%, respectively.^105,127^ The total cost per kW h (∼450$) is below other deployed battery systems including LIB, Pb-acid and redox flow cells and somewhat close to the above ground CAES technology.^108^

The total installed cost lies between $3000 and $4000 per kW of capacity and is comparable to that of LIB technology, *i.e.* 1800 and $4100 per kW of capacity.^128,129^ Taking into account the US DOE Energy Storage Database and the leader of the NGK Insulators, the >200 deployed systems^130,131^ yield roughly 584 MW of stored energy, suitable for up to 8 hours of daily peak shaving and a storage capacity of 3700 MW h, yet the number is more within the 4 GW h area.^17,131^

### 4.2 IT NaS battery

Reducing the operating temperature of the battery will lead to cheaper material costs associated with low cost polymeric seals and cell packing and mitigate thermal management issues such as heat loss that could enhance cycle life. In addition, organic solvents at lower temperatures could potentially access a greater portion of the theoretical energy density of the Na_2_S since the high melting point of the polysulfides limits the stoichiometric window of sodium to sulfur to ∼0.66.^48^ This thinking led to the development of the IT NaS battery, firstly introduced from Abraham in the early 80's^132^ and NASA.^116^

A number of studies on the IT NaS energy storage system using non-aqueous or polymer electrolytes have been reported, highlighting the increasing interest on this battery system^28,133,134^ The latest addition on this field entails a IT semi-flow lab-scale NaS battery having at the cathode a semi-solid suspension nanoscale carbon mixed with solid sulfur in a sodium iodide (NaI)/tetraethylene glycol dimethyl ether (TEGDME) solution.^22^ The effect of reduced operating temperature has been also demonstrated on the ZEBRA battery^89,135^ where a decrease of the operating temperature by 35 °C (from 275 to 240 °C) contributed in a 49% reduction in heating energy.

**Fig. 8:**
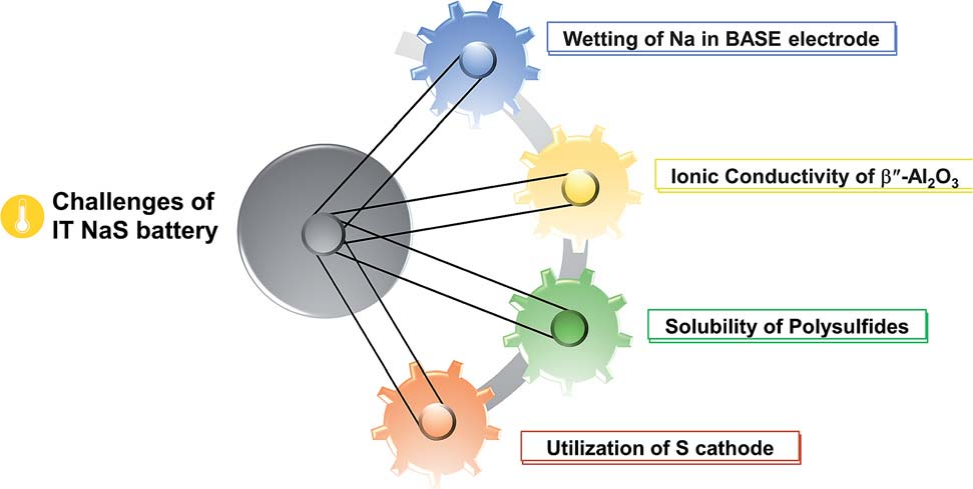
Challenges for the IT NaS battery system.

Regarding the challenges of the IT NaS system (Fig. 8), the high concentration of sodium is essential since the majority of the current is carried by the sodium cations and the transference number for sulfide ions is very small in melts of uniform composition.^38,136^ Secondly, the ionic conductivity of the ceramic electrolyte drops considerably at temperatures below 250 °C (*i.e.* < 0.2 S cm^−1^) leading to low power densities that are limited by the resistivity of the BASE. Thirdly, the solubility of sodium polysulfides in organic electrolytes is compromised at these lower temperatures. The density of higher order polysulfide melts increases with the decrease of temperature (*i.e.* 1.83 and 1.74 g cm^−3^ for *x* = 4 and 5, respectively) while irreversible formation of lower polysulfides (*x* = 2 and 3) and sulfur gives rise to capacity fade and cell failure.^137^ The last technical challenge involves the poor utilization rate of the sulfur cathode in the course of the electrochemical cell reactions. This issue is strongly correlated with the limited solubility of sodium polysulfides at this temperature range. The design of high-performance sulfur cathodes is rather challenging due to the delicate formulation between high loading, suitable electron conductivity and energy density.

Clearly, there are many challenges to be addressed here. Employing a thinner electrolyte to decrease resistance is imperative as well as optimizing electrode chemistry to improve electrochemical activity, especially on the cathode. For the design of reliable metal–sulfur batteries and for the detailed understanding of their performance, the need to employ advanced diagnostics for material and cell characterization, both *ex situ* and *in situ* is indispensable. Impedance spectroscopy, X-ray diffraction, Raman and UV-Vis spectroscopy, nuclear magnetic resonance as well as computational *ab initio* crystal-structure (CSP) and DFT first principle calculations have been implemented in order to detect both the stable and metastable phases of polysulfides during the operation of the battery cell. The above techniques are able to distinct and quantify the components of the polysulfide melt samples with great efficiency and success. A more detailed discussion of the fundamental technical challenges is provided below.

#### 4.2.1 Wetting of sodium on the BASE

**Fig. 9:**
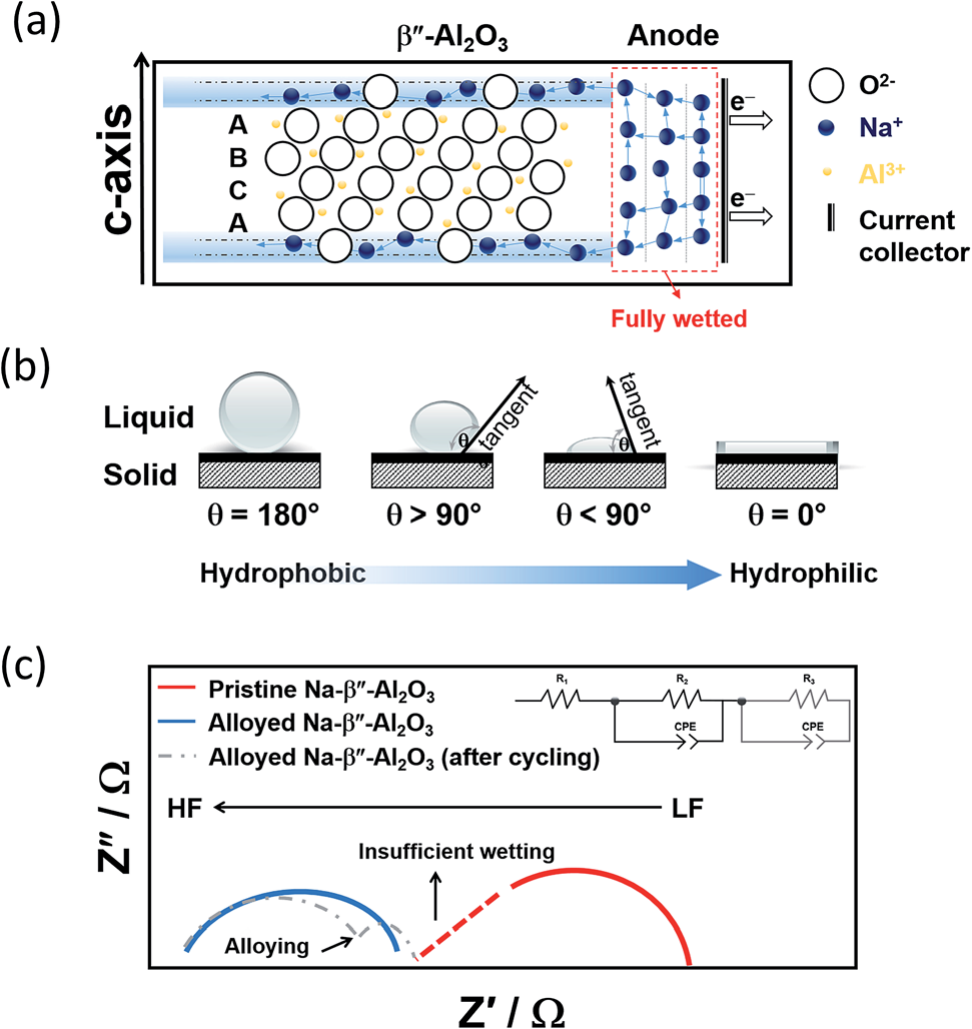
(a) Graphic depicting the wetting of Na-BASE at the IT range. The diagram is adapted/reproduced from ref. 67 and 141 with permission from *Elsevier*. (b) Schematic showing the different types of contact angles (c) Nyquist plots of Na symmetric cells showing the effect of alloying in the bulk (*R*_1_) and interfacial resistances (*R*_2_ and *R*_3_, the latter due to alloying). The diagram is adapted/reproduced from ref. 143 with permission from *Springer Nature.*

The wetting of the beta-alumina by liquid sodium is a solely temperature related issue with elevated temperatures (>300 °C) greatly facilitating it, as depicted in Fig. 9(a).^63^ When the NaS battery is assembled having sulfur in the cathode compartment, a high internal impedance is to be expected until discharge of the cell causes a significant amount of the highly conducting ionic liquid to be formed between the BASE and the cathodic current collector. High local current densities can occur with ineffective wetting, leading to degradation of the alumina tube under electrolytic conditions. To a certain degree, an effective way to improve wettability at lower temperatures requires the introduction of an intermediate metal layer to promote sodium wetting on the BASE at the desired temperature range. In general, metals with high conductivity such as sodium (*ca.* 2.1 × 10^7^ S m^−1^), have higher surface energy and are more readily wetted by molten metal than other materials.^138^

Up-and-coming examples as anode materials for the IT NaS implicate the combination of the BASE electrode with alloying metals such as Ni, Bi, Sn, Pt, In, Pb and mixtures like Na_15_Sn_4_.^98^ Normally, a 50–100 nm thick layer is introduced through sputtering or template and screen printing methods. These metals depending on their solubility with sodium, can be present in solid form or partially dissolved. Mechanical alloying has the distinct advantage of an inert and controlled atmosphere RT treatment.^139^ The removal of water through vacuum further improves the effect of the alloying.

A figure of merit to inspect the wetting of the BASE are contact angle measurements as described in Fig. 9(b). By definition, there is wetting of the surface if the contact angle is smaller than 90°. Angles greater than 90° indicate insufficient wetting. A number of studies on the alloying of the BASE have reported noticeably reduced contact angles, corroborating the improved wetting at temperatures between 105 and 180 °C.^140,141^ The alloying metals possess a larger surface tension than molten sodium, which in turn leads to stronger adhesion between solid and liquid and lower overpotentials during galvanostatic cycling. The interfacial resistance at the Na|BASE interface is greatly lowered as depicted from impedance measurements leading to superior cycling performances, especially in the cases of Bi and Sn.^98,142^ The semi-circle of Fig. 9(c) that is observed in the high frequencies (HF) of the Nyquist plot is ascribed to the bulk resistance (*R*_1_). If the wetting of the BASE electrode is insufficient, a depressed HF section of the semi-circle is evidenced. The interfacial resistance (*R*_2_), according to the equivalent circuit takes place between medium and lower frequencies (LF). The introduction of a constant phase element (CPE) models the inhomogeneities of the BASE surface. Depending on the alloy utilized, the appearance of a second semi-circle in the LF region depicts the interfacial resistance of the alloying metal and/or the diffusion of metal ions.^44,51,98^

Evidently, alloying can ameliorate the intrinsic safety of the battery by passivating the surface layer. Still, adherence seems to be the main concern and therefore the stability is under scrutiny since the dissolved alloyed metal can be detrimental to the electrochemical reactions of the cell. The introduction of a platinum grid on the BASE surface has been reported to lead to greater distribution of sodium melt on the active BASE surface area improving the wettability and in turn the cycleability of the cell.^91,144^ Other alloying metal electrodes tested at temperatures as low as 95 °C include Na–K and Na–Cs (both in 4 : 1 mole ratio) and Na–Rb (3 : 7 mole ratio). Na–Cs exhibited promising results when cycled at 150 °C with a capacity fade of 3% over 100 cycles (∼420 mA g^−1^), attributed to the lower surface tension of liquid Cs and stronger interaction between the latter and beta alumina atoms than those with sodium.^38,145^ Yet, taking into account the high cost of this particular metal (*viz.* $1100 per 100 g),^146^ even a content as low as 7 wt% renders it non cost-competitive. To conclude, alloying seems to effectively eliminate the interfacial effects and yield promising results, yet in a limited timeframe.

Aside from alloying, another attested way to improve the mechanical, electrical and magnetic properties of ceramics comes through doping. There is an ongoing research on ways to improve the beta alumina phase content, microstructure, mechanical strength and ionic conductivity. Several transition metal cations such as Ni^2+^, Co^2+^, Cu^2+^, Zn^2+^ Mn^2+^ and Ti^4+^ have been reported to enhance the ionic conductivity of the electrolyte.^147,148^ There is a critical ionic radius (*ca.* 0.97 Å) upon which the cation can enter the spinel block (illustrated in Fig. 5(b) and (c)) and replace the Al^3+^, thereby stabilizing the β′′-Al_2_O_3_.^149^ Cations with larger ionic radii such as Ag^+^, Ba^2+^, Sr^2+^ can enter the conduction planes and replace Na^+^, deteriorating the overall structure. The same can occur with water molecules.^150^ Recently, 1.0 wt% of Nb_2_O_5_ was found to improve the bending strength and ionic conductivity of the BASE.^151^ ZrO_2_ (<15% wt) has also been successfully incorporated into the β′′|β-Al_2_O_3_ matrix minimizing its resistivity^152^ while the interfacial resistance has been reported to improve through the introduction of coatings such as nickel that contained intertwined porous nanowires of ∼100 nm diameter.^141^

Other strategies towards the formulation of a robust BASE involve the addition of cationic surfactants such as cetyltrimethylammonium bromide (CTAB) on the β′′-Al_2_O_3_ nano-powder. Through a sol–gel process it was found to enhance the conductivity of the β′′-Al_2_O_3_ phase.^153^ In addition, the introduction of a bi-layer BASE that consists of a thin dense layer and a relatively thicker porous layer on a IT planar Na-MH battery (190 °C) was reported to enhance its energy efficiency for 350 cycles.^107^ The modified BASE contains a porous layer with micro-porosities facing the molten sodium that facilitates its movements and reduces the overall resistance.

The effect of Li^+^ and Mg^2+^ stabilizers on the β′′-Al_2_O_3_ phase content is also critical. The stabilization of the phase entails the substitution of the Al^3+^ ions by the cation of stabilizers, thereby compensating the extra Na^+^ ions (Fig. 5(c)).^154^ The Mg^2+^ ions substitute the tetrahedral Al^3+^ in the spinel blocks and the Li^+^ substitute the octahedral Al^3+^. The analogy between the octahedral and tetrahedral Al cations and the β and β′′-Al_2_O_3_ phases can be (a) tuned so as to reach greater conductivities and stabilities and (b) monitored easily since the relative amounts of β and β′′-Al_2_O_3_ phases are portrayed by their own characteristic diffraction peaks.

#### 4.2.2 Ionic conductivity of BASE at IT

**Fig. 10:**
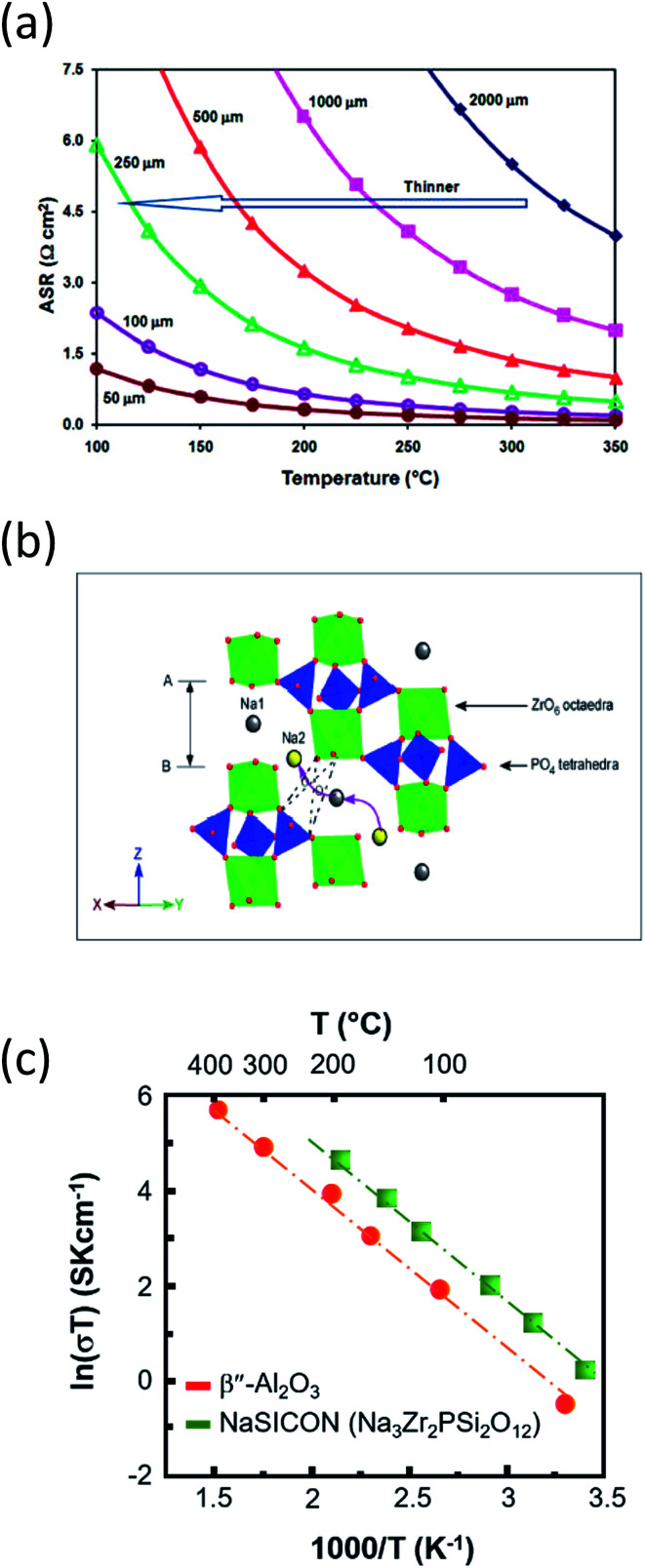
(a) Area specific resistance of BASE as a function of temperature and thickness. The diagram is adapted/reproduced from ref. 62 with permission from the *Royal Society of Chemistry*. (b) NaSICON structure in rhombohedral symmetry showing ZrO_6_ and (Si,P)O_4_ polyhedra. Curved arrows depict the conduction path of sodium ions. The diagram is adapted/reproduced from ref. 29 with permission from the *Royal Society of Chemistry*. (c) Temperature-dependent ionic conductivity of Na-BASE and Na super ion conductor (NaSICON). The diagram is adapted/reproduced from ref. 48, 180 and 181 with permission from the *Electrochemical Society*.

The BASE layer thickness is strongly linked with the area-specific resistance of the cell (ASR, Ω cm^2^). The smaller the thickness, the lower the ASR, which consecutively will lead to greater current densities employed on the system, especially on the sulfur cathode as upon successful mitigation of the interfacial resistance of the Na|BASE, the sodium electrode can introduce current densities of the order of 100 mA cm^−2^.^49^ From Fig. 10(a), it is clear that thicknesses smaller than 100 μm could lead to non-operable cell resistances since there is not enough flexural strength for the BASE to function. Scarce reports on relatively thin solid electrolyte membranes (154 μm) have been documented on HT SIB systems (350 °C) with moderate success though due to the low self-discharge rate and high tolerance to thermal cycling of the cell.^155^ In this context, a great deal of research has been conducted on devising ways to improve the conductivity of the BASE. The majority of these strategies have been deployed for RT NaS, IT ZEBRA and SIB systems and involve the introduction of polymers. Examples include polyethylene oxide with low-cost porous iron oxide films,^156^ polyvinylpyrrolidone-based surfactant with a Mg^2+^ stabilized Na-β′′|β-Al_2_O_3_ ^157^ and polyvinylidene fluoride.^158–160^ Apart from polymers, the densification of the Na-β′′|β-Al_2_O_3_ structure could be achieved with metal oxides such as TiO_2_ and ZrO_2_.^161^

Deploying superionic sodium conductors such as NaSICON (Fig. 10(b), Na_1+*x*_Zr_2_Si_*x*_P_3−*x*_O_12_ (0 ≤ *x* ≤ 3)),^162–167^ reinforced β′′-Al_2_O_3_ and yttria-stabilized zirconia (YSZ)^168–170^ Na_2_M_2_TeO_6_ (ref. 171 and 172) Na_3_PS_4_,^173,174^ Na_2_SMS_2_ (M = Si, Ge and Sn),^139,175^ alkali sulphide glass electrolytes^176,177^ and TiN based porous electronically conductive membranes that are selective to faradaic reactions instead of regulated ionic conduction^178^ have been reported as an alternative to BASE on an experimental stage. The above sodium ion conductors are primarily destined for solid state batteries, seeking to minimize the ohmic resistance between 0.01 and 0.5 S cm^−1^ at the set temperature and no capacity retention and shuttle phenomena.

The challenged ionic conductivity of NaSICON at the IT (Fig. 10(c)) is attributed in large to the presence of ZrO_2_ impurities upon synthesis of this superionic conductor. Luckily, the NaSICON framework is prone for a wide range of chemical substitutions and its mixing with beta alumna can lead to enhanced conductivities. A new group of P2-type phases namely Na_2_M_2_TeO_6_ (M = Ni, Zn, Co, Mg, *x* = 2/3) are stoichiometric, ordered and exhibit a sodium ion conductivity of the order of 4–11 S m^−1^ at 300 °C but still not as high in the IT and RT range. The crystalline glass ceramic Na_3_PS_4_ opened new possibilities for Na based conductors since the reported RT conductivity was similar to the one of NaSICON (*i.e.* 2.62 × 10^−4^ S cm^−1^).^173^ Other promising sodium conductors include doped sodium hybrids such as Na_2_B_10_H_10_, chalcogenide glass ceramic electrolytes (Na_2_Ga_0.2_Ge_1.8_Se_4.95_) and polymer electrolytes such as PEO–NaClO_4_–TiO_2_ where their RT temperature conductivities linger between 10^−6^ and 10^−5^ S cm^−1^.^179,180^ As a final point, the porous electronically conductive membranes can achieve chemical selectivity by preferred faradaic reduction of transition-metal ions such as Pb, Li and potentially Na, rather than by regulated ion conduction and can be used in all-metal displacement cell equipped with molten salt electrolyte yielding high current density and coulombic efficiencies (*i.e.* 92% at 150 mA cm^−2^ and *T* = 410 °C).^178^

All the above alternative sodium conductors show promising results, but are far away from deployment. The widely standardized method to evaluate the conductivity of the glass–ceramic electrolytes is electrochemical impedance spectroscopy. The high frequency region of the Nyquist plot reveals crucial information regarding the grain boundary resistance, that is the densification of the pressed bulk.^182^ Analogous Nyquist plots to the one of Fig. 9(c) are produced in such cases. In addition, a detailed understanding of such materials requires information on the atomic structure, because ionic transport properties are related to the environment of the mobile ions and the network structure. Therefore, neutron and X-ray diffraction measurements are used in several occasions.^182^

#### 4.2.3 Physicochemical characterization of IT NaS cell

One of the main components of the IT NaS cell entails the sulfur cathode and the formation of polysulfides. Sulfur has proved to be very amenable to molecular characterization by Raman spectroscopy. Yet, synthesizing pure samples of sodium sulfide (Na_2_S) is not an easy task due to the oxidative nature of these compounds. NaS_*x*_ melts are easily supercooled and glass forming at *T* > 400°. The decrease in the cell operating temperature creates a rather inflexible polysulfide chemistry. The structures of the most stable crystalline polysulfide phases displayed in Fig. 4(d), namely α-Na_2_S_2_, R-Na_2_S_4_ and R-Na_2_S_5_, are determined through experimental and theoretical experiments. Some metastable phases of these polysulfides including S_2_^2−^, S_3_^2−^ and S_5_^2−^ have been described through Raman spectroscopy,^183,184^ X-ray diffraction^185^ and ^23^Na solid state nuclear magnetic resonance (NMR) spectroscopy coupled with crystal-structure prediction algorithms (USPEX) and predicted theoretically through *ab initio* calculations.^75^ Regarding NMR, the values of the isotropic chemical shifts when coupled with the quadrupolar coupling constants and asymmetry parameters of the crystalline sodium polysulfide structures can lead to a reliable assignment of the ^23^Na crystallographic sites.^75^ Furthermore, an *in situ* spectro-electrochemical study in a dipolar aprotic media dimethyl sulfoxide (DMSO) confirmed the presence of the metastable phases (S_2_^2−^, S_3_^2−^ and S_4_^2−^ and S_6_^2−^ structures) that take the linear form whereas those of elemental S and S_8_^2^˙ are cyclic and less stable leading to a ring-opening reaction in search of a more stable structure.^186^

The lion's share of the majority of *in situ* and *ex situ* spectro-electrochemical studies are addressed for the RT LiS cell, aiming to detect the nature of polysulfides dissolved in the electrolyte.^187–189^*In situ* UV-Vis spectroscopy in DMSO, tetrahydrofuran (THF) and dimethylformamide (DMF) for the LiS cell showed that long chain polysulfides such as S_8_^2−^ and S_6_^2−^ are produced in the first reduction step due to the opening of the S_8_ ring. Shorter chain polysulfides like S_3_^2−^, S_2_^2−^ and S_2_^−^ appear at the end of the reduction process.^94^ Even though the chemistry and interaction of polysulfides with lithium and sodium is different, the shuttling of the charge carriers from the anode to the cathode is similar. Capitalizing on the above studies could potentially shed light on the structure of the sodium polysulfides on the IT range.

The majority of Raman studies on sodium polysulfides involve either solid or aqueous polysulfide samples, focusing largely on Na_2_S_4_.^190,191^ Janz and his coworkers conducted an in-depth Raman and infrared technique study to detect sulfur-containing anions in inorganic polysulfides including S_2_^2−^, S_4_^2−^ and S_5_^2−^ in aqueous electrolytes as well as crystalline sodium polysulfides.^183^ In all crystalline sodium polysulfides (Na_2_S_*n*_, *n* = 3–5), sulfur appears in the form of unbranched S_*n*_^2−^ chain-like ions. The negative charge of the S_*n*_^2−^ ion is compensated by the positive charge of two sodium ions. The hexagonal structures of sodium disulfide (α-Na_2_S_2_ and β-Na_2_S_2_) with three formula units per unit cell contain disulfide anions with sulfur bonds of length 215 pm. The crystals of α-Na_2_S_5_ and recently reported ε-Na_2_S_5_ are orthorhombic and contain anions of bend (*cis*) conformation and stretched symmetry (*trans* conformation), respectively.^75^ However, both are not thermodynamically stable as they transform into Na_2_S_4_ and sulfur at elevated temperatures (>250 °C). For Na_2_S_5_ melts with *n* > 3.6, the band of the S_5_^2−^ anion is dominant in the Raman spectra.^192–194^ The majority of the S–S stretching bands discussed above can be quantitatively and qualitatively identified between the 400 and 500 cm^−1^ band region.^195^

Another region of interest includes the interaction of Na_2_S and Na_2_S_2_ with different amounts of sulfur throughout a range of temperatures starting from 25 °C all the way to 400 °C. Solid Na_2_S initially transforms into crystalline R-Na_2_S_4_ having α-Na_2_S_2_ as an intermediate whilst complete sulfur melt occurs due to polymerization-depolymerization of small rings such as S_6_, S_7_ and S_8_ chains. Between 120 and 170 °C, the Raman spectra band positions change notably. In general, polysulfide modes are found between 100 and 800 cm^−1^ are associated with S–S bending and stretching variations. At 120 °C, the main bands of sulfur (152, 218, and 471 cm^−1^) disappear and new crystalline α-Na_2_S_2_ and R-Na_2_S_2_ peaks come about.^193^ The intensity of the R-Na_2_S_2_ peaks diminishes at temperatures between 140 and 160 °C, giving rise to α-Na_2_S_2_ through a conversion mechanism.^117^ Furthermore, at the IT range, the mixtures of Na_2_S with sulfur lead to the formation of R-Na_2_S_4_ as a crystal in equilibrium with the intermediate formation of sodium disulphide at temperatures lower than 160 °C.^193^

**Fig. 11:**
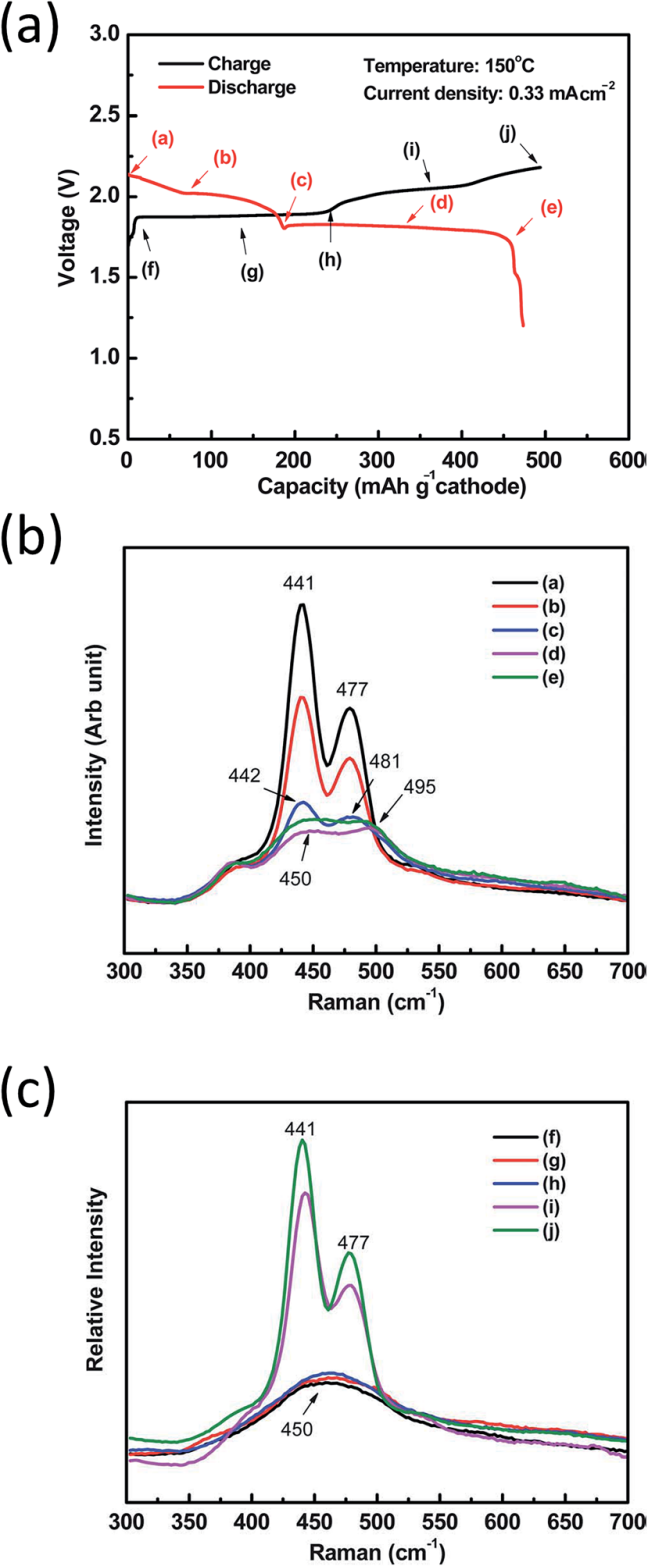
(a) Initial charge discharge plot of an IT planar NaS cell. Raman spectra of the IT NaS battery cathode during the first discharge (b) and charge (c) at a current density of 0.33 mA cm^−2^. The diagram is adapted/reproduced from ref. 28 with permission from the *Royal Society of Chemistry*.

The above solid samples are well established in good agreement with recent studies on NaS cells using (polar) organic solvents. More precisely, *in situ* Raman spectroscopy on the initial discharge–charge cycle of both IT planar and tubular NaS cells revealed main bands at 441, 477 and 491 cm^−1^ attributed to α-Na_2_S_5_, S and Na_2_S_4_, respectively, in line with the findings on crystalline sodium polysulfide samples.^28,91,190^ During the initial discharge of the cell (Fig. 11(a) from a–e), the reduction of elemental sulfur to dissolved polysulfides followed by a cascading conversion to lower ones is observed through the broadening and shift of the bands to the high-wavenumber side. Throughout higher depths of discharge, the sulfur lattice line at 477 cm^−1^ disappeared to give rise to another line at 495 cm^−1^, ascribed to Na_2_S_4_. The same occurred for α-Na_2_S_5_ (441 cm^−1^), which shifted rightward to 450 cm^−1^ showing lower Raman intensity and suggesting that lower non-soluble polysulfides are formed such as Na_2_S_3_ and Na_2_S_2_ following the reduction of α-Na_2_S_5_. The polysulfide species were formed in the following sequence during discharge: Na_2_S_5_ + S → Na_2_S_5_ + Na_2_S_4_ → Na_2_S_4_ + Na_2_S_2_ (Fig. 11(b)). From Fig. 11(a), during charge (from f–j) the reverse behavior is evidenced. The broad band at 450 cm^−1^ splits into two sharper ones indicating the formation of elemental sulfur (441 cm^−1^) and Na_2_S_4_ (477 cm^−1^) as shown in Fig. 11(c).^28^

The above findings can undeniably validate the reversible opening and reforming of the S_8_ ring during cycling in a similar manner to the LiS battery.^188^ Since it has been attested that Raman spectroscopy can detect the chemical species formed in the non-aqueous liquid state, it can be implemented in the monitoring of the electrochemical processes taking place during the operation of the battery that however has to be planar. Consecutively, this will help investigate more in depth the solubility of the electrolyte as thoroughly discussed in the section below.

#### 4.2.4 Solubility of sodium polysulfides at IT

Prior to determining the solubility of the sodium polysulfides at IT, the appropriate solvent has to be selected. The desired electrolyte should ensure high sodium ion conduction whilst acting as a poor solvent for the redox-active species present at the sulfur cathode. Sulfur due to its hydrophobic nature is soluble to nonpolar solvents such as toluene whereas lower order polysulfides (Na_2_S_*x*_, 0 < *x*< 2) are soluble in polar solvents like water. A thorough investigation on different classes of organic electrolytes was conducted several decades ago by NASA.^49^ Alcohol amines, amides, cyclic alcohols, and cyclic polyalcohols gave the most promising results in terms of solubility (0.5 to 2 M) and stability (*i.e.* no H_2_S and residue formation). Carbonate based electrolytes due to their low solvation power and high volatility and flammability (flash points between 16–33 °C for linear carbonates) are not as suitable electrolytes for the NaS system compared to glymes.^196,197^

The latter possess a greater solvation power as well as higher boiling and flash points than other solvents rendering them more suitable candidates.^198^ Their structure is unique since they contain multiple ether-type oxygen atoms and flexible alkoxy chains. The prevailing non-aqueous solvent used in the IT NaS is TEGDME stemmed from the on-going work on RT NaS batteries, the early feasibility study of Abraham, the more recent one of Ahn^132,134,199^ and on parallel work conducted on RT LiS and SIB.^134,200,201^ As all members of the glyme family, TEGDME is a stable and amphiphilic solvent and exhibits a higher boiling point (275 °C) compared to triglyme and butyl diglyme. It provides a large electrochemical window ranging from 0 to 3.5 V *vs.* Na|Na^+^ and in the presence of supporting salts such as NaI, NaPF_6_ and NaClO_4_ the window narrows down to approximately between 1 and 2.5 V.

**Fig. 12:**
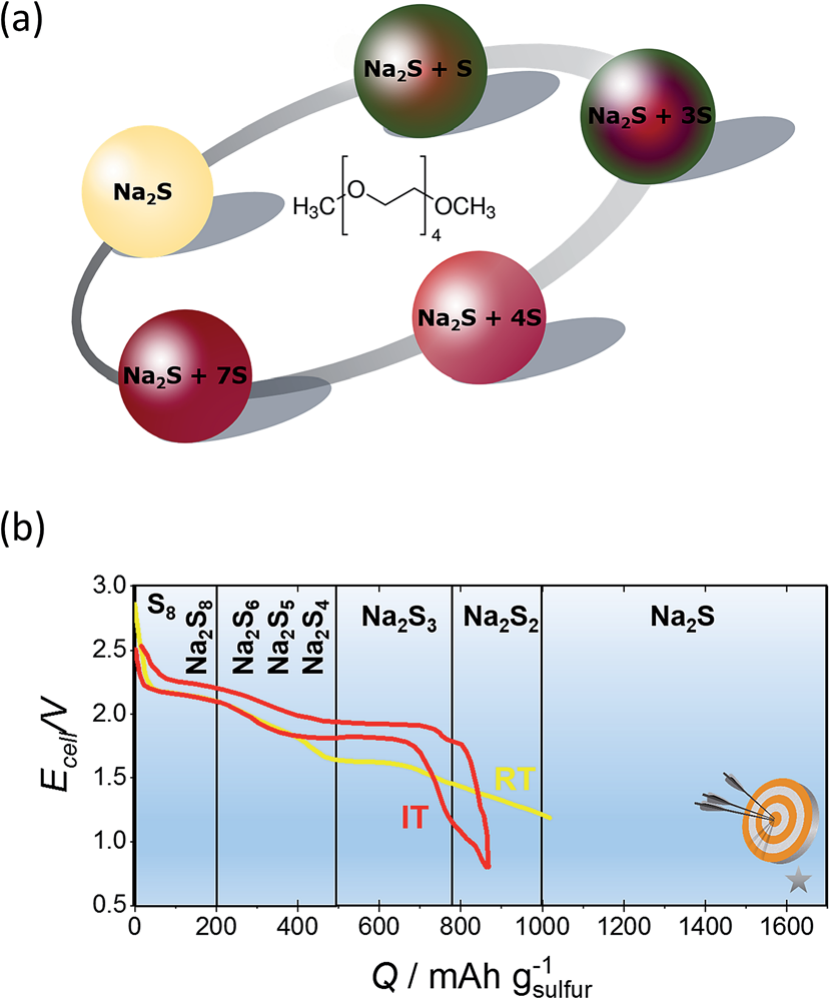
(a) Graphical representation of the colour of different sodium polysulfides when dissolved in TEGDME. (b) Initial charge–discharge curves of a NaS battery at IT and RT. The diagram is adapted/reproduced from ref. 30 with permission of the *Royal Society of Chemistry* and from ref. 212 with permission of *John Wiley and Sons*.

Sulfur reduction in non-aqueous electrolytes takes place *via* transformation of long-chain to short-chain polysulfides, accompanied with visible color changes since long-chain polysulfides adsorb light at higher wavelengths compared with short-chain ones.^188,202–204^ The color of Na_2_S solutions displayed in Fig. 12(a) varies markedly with the amount of sulfur dissolved in Na_2_S is colorless and becomes pale yellow upon the addition of a small amounts of elemental sulfur. The lower order polysulfides shift from transparent green (Na_2_S_2_) to yellowish green (Na_2_S_4_). As more sulfur is added, the solution (Na_2_S_5_) turns progressively darker, having a dark brown colour.

Regarding the performance of the IT cell, the semi-solid IT NaS battery when cycled between 2.5 and 1 V for 1 M sulfur nominal concentration in a solution containing TEGDME and 1 M NaI and at discharge currents of the order of 0.5 mA, could yield a storage capacity of 864 mA h g^−1^, which corresponds to the formation of Na_2_S_2_ (ref. 22) as seen from Fig. 12(b). In addition, a tubular IT NaS cell operating at 150 °C and a concentration range of 1.5 to 3 M Na_2_S_5_ dissolved in TEGDME, showing a robust long term performance (42 days of continuous cycling) with a volumetric energy density of 83 W h L^−1^ and capacity of 200 mA h.^91^

For the Na|TEGDME system, the electric current occurs from to the movement of free ions and the relative movement of ions within loosely bound ion pairs.^205^ The complexation of Na|TEGDME is assumed to be favourable and results in the formation of crown ethers that are well known to form stable complexes. The TEGDME molecules (having 4 oxygen atoms per molecule) tightly embrace the sodium ions within the solvent.^206^ Due to the large size of the molecule, there is a fraction of sodium that is not solvated by TEGDME and is attached to sulfur leading to the breaking/formation of long chain polysulfides *via* S_8_ ring opening during the oxidation/reduction processes. Sodium atoms have purely ionic nature like sodium cations in the Na_2_S crystals.^46^ Typically, small ions contribute to a greater extent to the conduction process leaving large and small ions responsible for diffusion and viscous momentum transfer.^207^

**Fig. 13:**
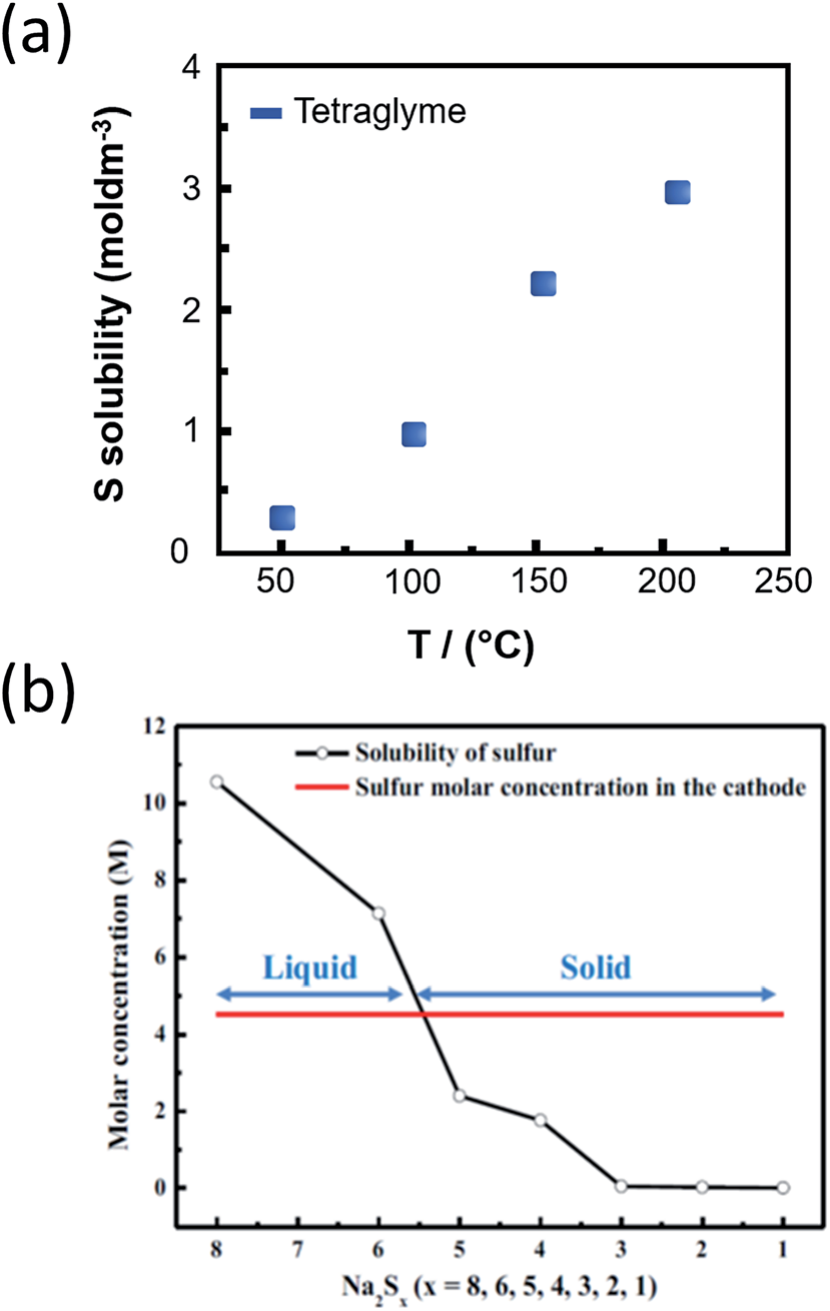
(a) Solubility of sulfur in TEGDME at the IT range. The diagram is adapted/reproduced from ref. 28 with permission from the *Royal Society of Chemistry*. (b) Relationship between the solubility of sulfur depending on Na_2_S_*x*_ (*x* = 1–8) and the sulfur cathode. The molar concentration of sulfur in the cathode is calculated as the sum of liquid electrolyte and sulfur mass in the cathode. The diagram is adapted/reproduced from ref. 209 with permission from the *Electrochemical Society*.

For the realization of practical IT NaS cell current densities, the discharge products should have adequate solubility (>1.5 M) to minimize the formation of resistive films by excessive precipitation of the sulfides on the BASE or on the current collector. The dissolution of sodium polysulfides in TEGDME, can be controlled by tweaking the sulfur to TEGDME weight ratio. Fig. 13(a) describes the relationship between temperature and solubility of sulfur in this polar aprotic solvent. A maximum solubility of 2.3 M is achieved at 150 °C.^28,208^ In contrast, the solubility of Na_2_S_5_ and Na_2_S is lower by two orders of magnitude. Higher order sodium polysulfides (*x* = 5–8) are viscous and highly soluble, reaching 10.5 and 2.4 M for Na_2_S_8_ and Na_2_S_4_, respectively. Fig. 13(b) sets the operational range regarding the polysulfide concentration, as lower order polysulfides (*x* = 1–3) are considerably less soluble and therefore would hamper the operation of the cell.^209^ All in all, the solubility in the IT NaS battery allows for competing energy densities that have to be closely monitored since upon yet formation of lower polysulfides the densities will fade dramatically.

#### 4.2.5 Kinetics and performance of the IT NaS battery

The performance of the IT NaS cell can be investigated through *in situ* techniques that give an indication of the formed species that occur during its operation. Other crucial parameters include the efficiencies of the cell involving the coulombic, voltaic and energy efficiencies as well as the kinetics of the system. The reported round trip energy efficiency of the HT NaS lingers above 80% (ref. 129) rendering very competitive amongst its peers. Naturally, the same efficiencies are to be expected for the IT NaS system, if not better.

**Fig. 14:**
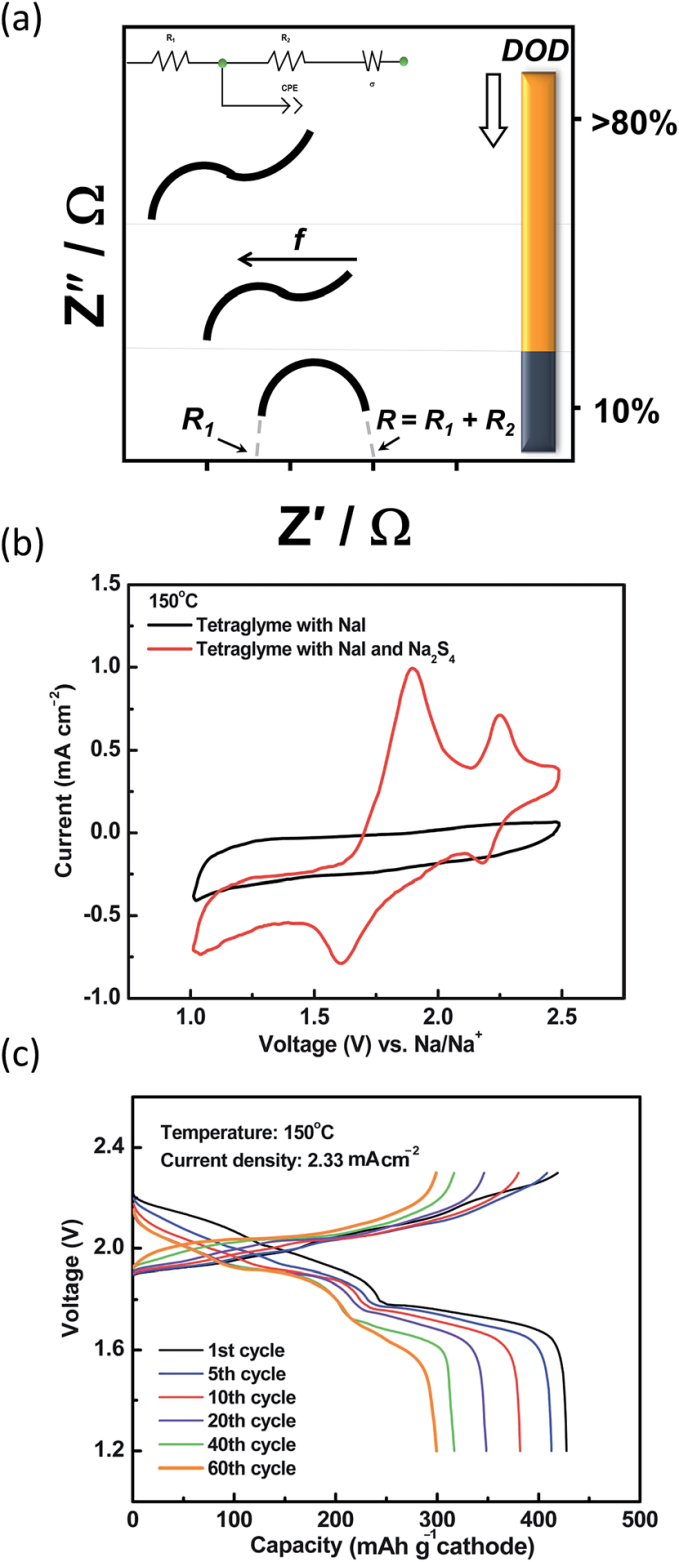
(a) Nyquist plots of polysulfide melts as a function of depth of discharge (DOD). The diagram is adapted from ref. 212 with permission from *Elsevier*. (b) Cyclic voltammetry of a planar IT NaS cell containing NaI, TEGDME and Na_2_S_4_ (scan rate: 5 mV s^−1^). The diagram is adapted/reproduced from ref. 28 with permission from the *Royal Society of Chemistry*. (c) Cycleability of a IT NaS planar cell (current: 7 mA). The diagram is adapted/reproduced from ref. 28 with permission from the *Royal Society of Chemistry*.

The techniques implemented to determine the kinetics of the reactions at the IT range include electrochemical impedance spectroscopy and polarization measurements. The impedance of molten polysulfide melt is commonly represented by a Randles circuit with a Warburg element present at the lower frequencies <10 mHz (ref. 210) as depicted in Fig. 14(a). The high frequency intercept gives the ohmic resistance (*R*_1_) of the cell (*i.e.* cell case, carbon electrode, BASE, electrolyte, leads and connections) and the middle frequency gives the polarization resistance of the cell (*R*_2_). At the low frequencies, the Warburg term describes the sodium diffusion or the sodium|electrolyte interface. At elevated temperatures, the lower polysulfides (*x* ≤ 3) are more conductive, shifting the ohmic resistance to lower values with increasing depth of discharge.^211,212^

Other techniques such as rotating disk electrode have also been reported. A study on a vitreous carbon rod in molten sodium polysulfides confirmed the presence of a diffusion controlled redox process near the open circuit potential. In the same study, at extreme positive and negative potentials from the open circuit potential, the appearance of sulfur globules and sodium polysulfide film occur, respectively.^80,213^ The diffusion coefficients (*D*) on polysulfide melts for Na_2_S_5_ were between 1 × 10^−5^ and 6 × 10^−7^ cm^2^ s^−1^ and for Na_2_S_4_*D* was reported equal to *ca.* 2 × 10^−7^ cm^2^ s^−1^.^79,80^ The cyclic voltammetry in the presence of NaI and 0.05 M Na_2_S_4_ in TEGDME confirmed the presence of two pairs of redox couples at 2.22 and 1.75 V *vs.* Na|Na^+^ representing the (quasi)reversible oxidation and reduction electrochemical reactions of the polysulfide species (Fig. 14(b)). Similar trends have been reported in another electrochemical study using *N*,*N*-diethylacetamide with 0.1 M NaBF_4_ as the supporting electrolyte.^28^ Interestingly, the cyclic voltammogram on a RT NaS cell using TEGDME, revealed two sharp and narrow cathodic peaks at 2.24 and 1.73 V, followed by two reverse anodic peaks at 1.94 and 2.32 V, respectively. The peak to peak separation (Δ*E*_pp_) from these two redox peaks is within the quasi-reversible range suggesting that the electrochemical reactions occur in a homogeneous manner and short voltage range.^209^ In all cases, the operational voltage window stemmed from the cyclic voltammograms was between 1 and 3 V.

Contrary to the well-defined quasi-reversible voltammograms, the cycling behavior of the cell is characterized with sizeable capacity loss upon a small number of cycles. Using TEGDME and NaI as solvent and supporting electrolyte, throughout 60 charge–discharge cycles (current density of 2.33 mA cm^−2^ normalized to the area of the cathode) the planar cell lost 30% of its initial capacity, falling to 300 mA h g^−1^. According to Fig. 14(c), the first discharge achieved was *ca.* 425 mA h g^−1^ corresponding to Na_2_S_4_. The coulombic and voltaic efficiencies lingered above 90% and 82%, respectively throughout the whole duration of cycling yielding an average energy efficiency of the order of 75%.^28^ The capacity loss largely stems from the irreversible dissolution and immigration of sulfur species in the liquid electrolyte as regularly diagnosed in RT NaS and LiS cells.^33^ The introduction of additives seems as the most direct way to mitigate the insolubility of the lower polysulfides. For example, a IT ZEBRA cell using a liquid alloy of Na–Cs (molar ratio of 1 : 4) and an ionic liquid (NaAlCl_4_) could get 200 reversible cycles at 2.33 mA cm^−2^ during a limited operational range (80% and 20%) so as to avoid propagation of the irreversible polysulfide formation. Furthermore, the recently reported carbon-less IT NaS tubular cell using 2.5 M Na_2_S_5_ in TEGDME showed good cyclic performance for 36 deep cycles with a capacity retention of 98%.^91^ Introduction of flow is expected to ameliorate the cycleability of the batter through enhanced mass transport effects.

Overall, the IT NaS system aspires to drive down the cell operating costs and create a safer environment. Yet, along with the reduced temperature, technical issues surfaced that have to be overcome in order to successfully demonstrate its economic and practical viability. Vigorous research on this and other inter-related fields have managed to sizably mitigate these issues, namely the ionic conductivity and wetting of BASE, solubility of polysulfides and utilization of the sulfur cathode. Nevertheless, more work is needed to get this system on par with the one operated at the HT range.

### 4.3 RT NaS cell

Decreasing the operating temperature of the NaS battery even further (25 °C) whilst maintaining the same performance metrics with the HT one (*viz.* round trip efficiency of 75% and calendar life of 4500 cycles) will be greatly welcomed in terms of safety and cost. Of late, there has been an increasing number of groups focusing on RT NaS battery attested by the increasing number of papers and reviews in this field.^29,30,33,48,214^ This trend has been in part initiated by the work conducted on RT LiS batteries as the technology and chemistry of the two electrochemical energy storage systems is alike.

Nonetheless, sodium atoms and ions are larger compared to lithium, (+82% for the atom and 25% and 55% for the ion, depending on the coordination) leading to larger volume changes during cycling.^48^ Interestingly, the difference in size between sodium atom and sodium ion is so large that when operated in RT conditions, sodium is more prone than lithium to form dendrites and unstable deposits.^215^

The chemistry and reaction mechanism of sulfur with sodium at RT is different from that at higher temperatures mainly due to the solid-state nature of sodium that leads to dendritic growth and to short circuit during charging. Besides, the sodium metal upon contact with the electrolyte forms a non-uniform solid electrolyte interface (SEI) layer that is ionically conducting but electronically insulating. Several strategies have been proposed to combat dendritic formation, stabilize the SEI and provoke fast electron transfer most of which stem from the Na-ion battery field.^216^

Regarding the material of choice at the cathode, the preparation of a flexible current collector is normally followed by its combination with a rigid active material. Numerous methods have been reported to accomplish the above, such as hydrothermal reaction, photothermal reduction, pulsed laser deposition, ultra-sonication and co-deposition, casting and drying.^27^ The combination of current collector with active materials can be avoided with the introduction of novel singled-body flexible cathodes solely consisting of the active material (*i.e.* sulfurized polyacrylonitrile nanofiber web, free-standing, binder-reduction, pulsed laser deposition, ultra-sonication and co-deposition, casting).^217^ A wide variety of carbon materials (1D, 2D and 3D) have been used as conductive frameworks in S/C composite electrodes with mesoporous carbon being the most attractive since their pores in their matrices can incorporate sulfur and provide both electrical conductivity and suitable space for the volume change (up to 74%) that occurs during the operation of the battery.^218–221^ Non-carbonaceous materials such as polyacrylonitrile-sulfur composites, metal oxides (TiO_2_, MoO_3_), disulfides (TiS_2_, ZrS_2_) and hydroxides (Ni(OH)_2_) have also been investigated as potential sulfur cathode electrodes aspiring to effectively trap the polysulfides.^222^

With respect to electrolyte formulation, polymer electrolytes have the advantage of ease of construction since they can be easily casted as thin films where the thickness can be regulated to accommodate the desired performance of the battery. Examples include polyethylene oxide (PEO), polyvinylidene fluoride (PVDF), poly(acrylic acid) (PAA) and carboxymethyl cellulose (CMC), gel polymers as well as organic solvents like ethylene carbonate, dimethyl carbonate and propylene carbonate.^219,223,224^ As an indication, a NaS cell below the melting point of sulfur using a PEO electrolyte showed a discharge capacity of 505 mA h g^−1^, in line with the theoretical value for complete discharge to Na_2_S_3_.^134^

In relation to the choice of an appropriate solvent, the solvation of sulfur with sodium ions varies with each solvent and therefore the exact mechanism of the multi-step reactions leading to the formation of polysulfides is still unclear. In the case of TEGDME, the low conductivity of sodium (*viz.* 10 × 10^−5^ S cm^−1^) and the high solubility of long-chain polysulfides in this solvent result in their migration from the cathode to the anode where they can undergo redox-reactions, *i.e.* shuttle effect. The latter leads to rapid capacity fade and low coulombic efficiency as well as loss of irreversible active materials. Yet, discharge capacities reaching 600 mA h g^−1^ have been reported with limited cycle life^225^ while lower ones (*i.e.* 250 mA h g^−1^) can be achieved with improved cycleability (500 cycles).^226^ A number of strategies have been proposed to mitigate the shuttling of the cell including the introduction of the “solvent-in salt electrolyte”, the embedding of sulfur species in a functional matrix, passivation of the anode and the altering the cell configuration.^33,227^

To sum up, the efforts in this field have been mainly focused on the development of functional nanocomposites utilizing efficient electrolytes and constructing novel cell configurations to obtain credible batteries. Most significantly, bettering the platting and stripping reactions of sodium metal along with the introduction of additives to mitigate the formation of dendrites and non-uniform SEI layer is another important aspect that certifies long cycle life. This RT battery technology does show great promise on becoming a reliable high energy density power source, yet its stability is currently compromised by the sluggish electroactivity, limited cycle life, rapid polysulfide migration and self-discharge. Capitalizing on the continuing and accumulated work in this field can help researchers to optimizing further the enabling components of the IT NaS battery.

### 4.4 Alternative technologies

There is a clear sense that the NaS chemistry in all temperature domains is getting better understood due to the numerous analytical and theoretical techniques that have recently flourished on this area. This is capitalized on other sodium-based technologies too. Another metal–chalcogen battery recently reported is the NaSe battery through the use of selenium/carbon tubes as a cathode. Paired with metallic sodium, this battery delivered a reversible energy density of 860 W h kg^−1^, normalized by the life of Se.^228^ Hybrid Na-based battery systems such as the NaS/NiCl_2_ are also an interesting alternative due to their superior energy density potential. The mixed NaS and ZEBRA chemistries in conjunction with a tailored cathode and electrolyte can operate at 280 °C, alleviating the corrosion of polysulfide melts and yield a 95% capacity retention over 60 cycles.^229^

The use of molten salts as secondary electrolytes in the ZEBRA cell has progressed markedly since the standard chloroaluminate electrolyte has been replaced by other alkali metal salts such as NaBr, LiCl and LiBr.^230^ Stable cell performance was evidenced at 150 °C on par with the normal ZEBRA cell operated at 300 °C. Another all-liquid metal battery is the NaZn free liquid metal battery. This low cost battery employs liquid sodium and zinc at the anode and cathode, respectively and a NaCl–CaCl_2_–ZnCl_2_ molten salt electrolyte.^231^ The flat discharge voltage of this battery was above 1.1 V when discharged at 100 mA cm^−2^, yielding coulombic efficiencies greater than 90%.

Shifting away from sodium to other affordable and environmentally friendly metals is perplexing, yet potentially rewarding in the long run. The logic behind the substitution of sodium lies in its reactive and unstable nature as well as its solvation and bonding with the solvent and polysulfides. Potassium (K), magnesium (Mg) and aluminum (Al) are suitable candidates in terms of cost and electrochemical properties to substitute sodium. Already, a novel potassium–sulfur (KS) battery with a K conducting BASE has been demonstrated.^138,222^ Replacing sodium with potassium in the anode can address the issue of ion exchange and wetting at lower temperatures, leading to greater energy efficiency gains.^232,233^ By using pyrolyzed polyacrylonitrile/sulfur as a positive electrode for RT KS battery, a reversible capacity of 270 mA h g^−1^ was achieved.^234^ The MgS battery based on a two electron conversion reaction of Mg can theoretically yield a volumetric energy density of 3833 mA h cm^−3^. Yet, the compatibility of the sulfur cathode with the scarce Mg ion conducting electrolyte hinders so far its progress.^235,236^ AlS batteries possess a high gravimetric capacity (*i.e.* 2980 mA h g^−1^), are dendrite free (*i.e.* high stripping/plating efficiency) and can utilize low materials cost due to the abundance of both active elements. The introduction of non-aqueous electrolytes however, creates significant issues on its long-term operation.^237^ The above proposed alternatives pave the way of forward innovative thinking that can revolutionize the molten metal based batteries and to a certain extent the whole energy storage outlook.

## 5 Conclusions

The significance of energy storage as a transformer of the way homeowners, businesses and utilities produce and use power is corroborated by the diverse set of companies including power providers, grid operators, battery manufacture and energy-storage integrators that have put energy storage a priority in their portfolio. The discussed HT and IT batteries can be competitive compared to other grid-scale electrical energy storage systems in both performance and cost. In terms of performance, the challenge lies to further accessing the theoretical energy density of the cell in a reversible and safe manner. Practical specific energies of the order of 250 W h kg^−1^ and specific power densities of 400 W kg^−1^ are a perquisite for instituting further the NaS technology.

**Fig. 15:**
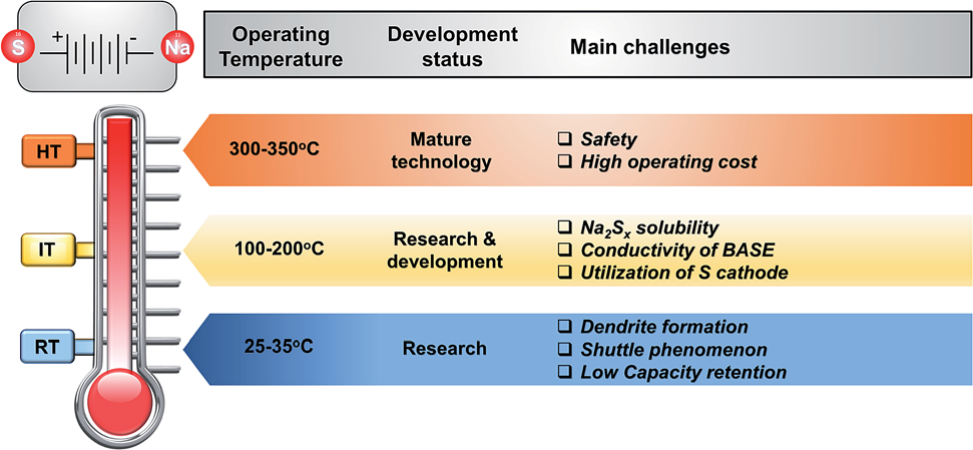
Summary and challenges of the NaS battery.

The HT system is well-established globally and currently competitive in the energy storage market. Nonetheless, the need to operate it in a more secure temperature environment makes sense in both safety and economic viewpoints (Fig. 15). A decrease in temperature will allow for improvement in materials durability and easier thermal management. Cost-effective sealing technologies through the use of polymer seals for the cell architecture can replace conventional expensive sealing methods including glass seals, TCB, and electron beam welding. The much needed reduction in the operating temperature leads to insufficient electronic conductivity and poor utilization of both anode and cathode making its robust performance a major challenge. Therefore, a multifaceted approach is needed to solve these issues. Efforts should focus on salt and solvent in the catholyte, employment of a suitable coating or dopant on the BASE in order to improve the wettability of molten sodium and the introduction of additives that can improve the performance of the battery by enhancing the polysulfide kinetics and solubility of sulfur (Fig. 15).

Furthermore, the IT NaS cell can use the know-how in terms of manufacturing, operating and monitoring of the HT system for its own commercialization purposes. Since the technologies are alike, the transition stage can be relatively smooth. The RT systems can also provide ideas and strategies related to the issues of the IT NaS cell. For example, in order to improve the ionic path for sodium migration at the metal/ceramic interface the application of a sodium ion conductive polymer interlayer at the sodium/solid interface is implemented^158^ that could easily be applied to the IT system too. What's more, the use of ionic liquids that has emerged from RT systems, stands as a reliable and safe electrolyte substitute for a number of battery systems^238,239^ due to their inert nature on parasitic reactions and sodium metal, good thermal stability, low flammability, tunable polarity and basicity/acidity large electrochemical window (>5 V) and comparable ionic conductivity (1 × 10^−2^ S cm^−1^ at RT^240^). Implementing novel electrolytes to the IT NaS is a plausible and interesting direction. Yet, there are several technical and economic challenges mainly related with the chemistry of the IL with polysulfides and the economic feasibility of the raw materials comprising the ILs.

Finally, despite the dominance of tubular HT NaS cells in the market currently, the use of planar ones should not be dismissed. The planar design offers flexibility in *in situ* monitoring techniques such as Raman spectroscopy and more importantly in the stacking of the electrodes, making it more space-efficient, thus leading to higher rate capability due to the larger active surface area for a given cell volume. In addition, thinner cathodes (<1 mm) can be introduced minimizing the cell polarization resistance and resulting in higher power output. Of late, there has been an increasing interest on planar Na-MH batteries.^229,241^

## Conflicts of interest

There are no conflicts to declare.

## Abbreviations

BASE: Beta alumina solid electrolyte
DFT: Density functional theory
NaS: Sodium sulfur
NaNiCl_2_: Sodium nickel chloride
IT: Intermediate temperature
RT: Room temperature
HT: High temperature
IL: Ionic liquid
TEGDME: Tetraethylene glycol dimethyl ether or tetraglyme
TCB: Thermal compression bonding
SEI: Solid electrolyte interface
RFB: Redox flow battery
LIB: Lithium ion battery
LTO: Lithium titanate
LCO: Lithium cobalt oxide
LMO: Lithium manganese oxide
LFP: Lithium iron phosphate
LiS: Lithium sulfur
SIB: Sodium ion battery
NaI: Sodium iodide
Na-MH: Sodium metal-halide
NMC: Lithium nickel manganese cobalt oxide
NCA: Lithium nickel cobalt aluminium oxide
Ni-Cd: Nickel cadmium
ZEBRA: Zero emission battery research activities

## Supplementary Material
